# Employment of patients with kidney failure treated with dialysis or kidney transplantation—a systematic review and meta-analysis

**DOI:** 10.1186/s12882-021-02552-2

**Published:** 2021-10-22

**Authors:** Lilli Kirkeskov, Rasmus K. Carlsen, Thomas Lund, Niels Henrik Buus

**Affiliations:** 1 Centre of Social Medicine, University Hospital Bispebjerg-Frederiksberg, Nordre Fasanvej 57, Vej 8, Opgang 2.2., 2000 Frederiksberg, Denmark; 2 Department of Transplantation Medicine, Oslo University Hospital, Sognsvannsveien 20, OUS, Rikshospitalet, 0372 Oslo, Norway; 3 Department of Public Health, University of Copenhagen, Copenhagen, Denmark; 4 Department of Renal Medicine, Aarhus University Hospital, Palle Juul-Jensnes Boulevard 35, indgang C, plan 2, 8200 Aarhus, Denmark

**Keywords:** Kidney failure, Renal failure, End-stage renal disease, ESRD, Haemodialysis, Peritoneal dialysis, Kidney transplantation, Renal transplantation, Employment rate

## Abstract

**Background:**

Patients with kidney failure treated with dialysis or kidney transplantation experience difficulties maintaining employment due to the condition itself and the treatment. We aimed to establish the rate of employment before and after initiation of dialysis and kidney transplantation and to identify predictors of employment during dialysis and posttransplant.

**Methods:**

This systematic review and meta-analysis were carried out according to the Preferred Reporting Items for Systematic Reviews and Meta-Analysis (PRISMA) guidelines for studies that included employment rate in adults receiving dialysis or a kidney transplant. The literature search included cross-sectional or cohort studies published in English between January 1966 and August 2020 in the PubMed, Embase, and Cochrane Library databases. Data on employment rate, study population, age, gender, educational level, dialysis duration, kidney donor, ethnicity, dialysis modality, waiting time for transplantation, diabetes, and depression were extracted.

Quality assessment was performed using the Newcastle–Ottawa Scale. Meta-analysis for predictors for employment, with odds ratios and confidence intervals, and tests for heterogeneity, using chi-square and I^2^ statistics, were calculated. PROSPERO registration number: CRD42020188853.

**Results:**

Thirty-three studies included 162,059 participants receiving dialysis, and 31 studies included 137,742 participants who received kidney transplantation. Dialysis patients were on average 52.6 years old (range: 16–79; 60.3% male), and kidney transplant patients were 46.7 years old (range: 18–78; 59.8% male). The employment rate (weighted mean) for dialysis patients was 26.3% (range: 10.5–59.7%); the employment rate was 36.9% pretransplant (range: 25–86%) and 38.2% posttransplant (range: 14.2–85%). Predictors for employment during dialysis and posttransplant were male, gender, age, being without diabetes, peritoneal dialysis, and higher educational level, and predictors of posttransplant: pretransplant employment included transplantation with a living donor kidney, and being without depression.

**Conclusions:**

Patients with kidney failure had a low employment rate during dialysis and pre- and posttransplant. Kidney failure patients should be supported through a combination of clinical and social measures to ensure that they remain working.

**Supplementary Information:**

The online version contains supplementary material available at 10.1186/s12882-021-02552-2.

## Background

Kidney failure with a need for renal replacement therapy affects approximately 0.1% of the global population. According to National Kidney Foundation statistics, more than 2 million people worldwide receive chronic dialysis treatment or are living with a functioning kidney transplant [1, 2]. Kidney failure reduces quality of life, increases psychosocial problems and has profound implications for the maintenance of normal employment [3, 4]. To a large extent, this is a consequence of disease-related comorbidity and uraemia-related symptoms, but it is also due to time-consuming treatments with haemodialysis or peritoneal dialysis. Therefore, kidney failure entails not only high costs because of the treatment itself but also results in lost productivity due to a reduced labour force. A Canadian study stated that kidney diseases cost more than 217 billion Canadian dollars annually in health care services alone [5]. In addition to this comes loss of labour force.

Over the past decades, replacement therapy in kidney failure has improved in terms of home-based dialysis modalities with automated peritoneal dialysis or home haemodialysis, rendering it easier for some patients to plan their time. Additionally, an increasing number of patients are receiving kidney transplants, and the survival rate following transplantation has increased [6]. Despite this, studies from all over the world have shown that many patients with kidney failure are not employed [7].

The employment rate in the general population of 15 to 64 years of age ranges between countries from 46 to 47% in South Africa and India to 85% in Iceland. The average employment rate in the Organization for Economic Co-operation and Development (OECD) countries is 69% [8]. The employment rate is lower in subjects below the upper secondary educational level than in those at or above the upper secondary level [8]. For subjects suffering from chronic diseases, the employment rate is lower. Prognostic factors for employment include severity of the chronic disease, employment status before getting the condition and educational level [9–11]. These somatic and social factors may also influence employment status in kidney failure patients.

Previous studies have reported employment rates and predictors for employment during dialysis or after kidney transplantation, but the results have never been summarized in a systematic review of kidney failure patients receiving dialysis or having a kidney transplantation [12–14]. The first aim of this study was to conduct a systematic review focusing on the employment rate before and after the initiation of dialysis (haemodialysis and peritoneal dialysis) and after kidney transplantation. The second aim was to establish predictors of employment during dialysis and posttransplant. The predefined predictors were socioeconomic factors, such as age, gender, level of education, and pretransplant employment, disease-related factors, such as dialysis modality, time on dialysis, waiting time for transplant, and donor type, and comorbidities, such as diabetes and depression.

## Methods

### Protocol

This systematic review was carried out according to Preferred Reporting Items for Systematic Reviews and Meta-Analysis (PRISMA) [15] for studies that included employment rate in kidney failure patients during dialysis and after kidney transplantation. The PROSPERO registration number is CRD42020188853.

### Selection criteria and search strategies

The literature search included the period from January 1966 to August 2020 in the PubMed, Embase, and Cochrane Library databases using the following search terms: ((chronic* kidney disease OR chronic* renal disease OR kidney transplant* OR renal transplant* OR dialysis OR hemodialysis OR peritoneal dialysis) AND (employment OR work ability OR disability pension)). Articles in English were included. The search was performed in the following order: PubMed, Embase, and Cochrane Library. Articles were selected primarily based on the titles and abstracts if necessary. Studies from around the world were included. Articles including employment, work ability or disability, return to work or disability pension were selected, and duplicates were excluded. Reference lists in the selected articles were reviewed, and more articles were included if relevant. Full-time and part-time employment, but not ‘working as housewives’, was included in our definition of employment.

### Data extraction, quality assessment and risk of bias

The data collected included author names, year of publication, study design, data collection dates, employment rate, study population, age, gender, educational level, dialysis duration, kidney donor, ethnicity, dialysis modality, waiting time for transplantation, diabetes, and depression. Quality assessment was independently assessed by two reviewers (LK and RKC) using the Newcastle–Ottawa Scale (NOS) for cross-sectional and cohort studies [16] to assess the risk of bias for all studies. Any disagreements were resolved by discussion until consensus was reached. The rating scale was based on 9 items that divided the studies into high (7–9), moderate (4–6) or low (1–3) quality. A low NOS score (range 1–3) indicated a high risk of bias, and a high NOS score (range 7–9) indicated a lower risk of bias. For cross-sectional studies, the quality assessment included representativeness of the sample, sample size, nonrespondents, ascertainment of the risk factor, comparability, assessment of outcome, and statistical testing. For cohort studies, the assessment included representativeness of the exposed cohort, selection of the nonexposed cohort, ascertainment of exposure, demonstration that the outcome of interest was not present at the start of study, comparability, assessment of outcome, length of follow-up and adequacy of follow-up.

### Analytical approach

For outcomes reported in numbers or percentages, odds ratios and 95% confidence intervals (CIs) were calculated if possible. Meta-analysis for the predefined predictors for employment before and during dialysis and after kidney transplantation, including age, gender, level of education, previous employment, dialysis modality, time on dialysis, waiting time for transplant, donor type and comorbidities such as diabetes and depression, were carried out. In addition to the predefined predictors, attempts were made to find information on ethnicity, health insurance, self-assessed ability to work and quality of life, but there were only enough data on ethnicity for analysis. Tests for heterogeneity was performed using chi-square and I^2^ statistics, where an I^2^ value below 40% might not be important, 30–60% might represent moderate heterogeneity, 50–90% represents substantial heterogeneity, and 75–100% indicates considerable heterogeneity.

Meta-analysis for predictors for employment, with odds ratios and confidence intervals, and tests for heterogeneity were calculated using Review Manager software (RevMan, version 5.3. Copenhagen: The Nordic Cochrane Centre, The Cochrane Collaboration, 2014).

## Results

### General description of included studies

**Fig. 1:**
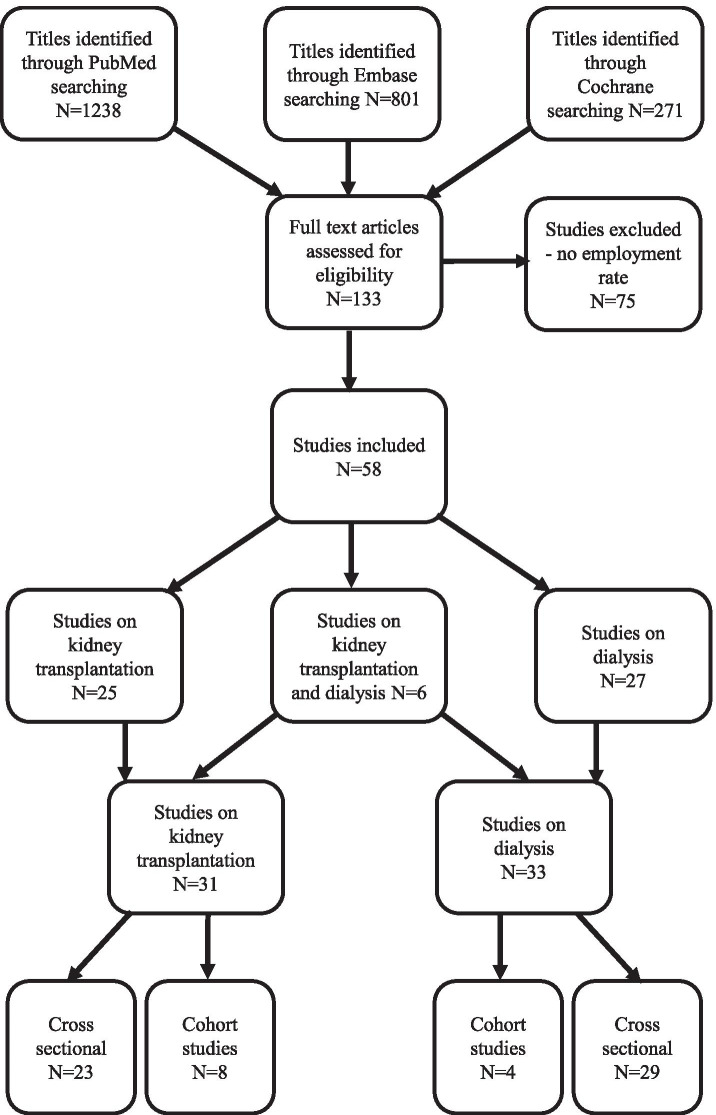
Flow chart illustrating the systematic search for studies examining employment outcomes in patients with kidney failure receiving dialysis or transplantation

The search yielded 2310 references addressing kidney failure and employment. From the titles, 133 studies were considered relevant for evaluation, and of those, 58 met the inclusion criteria. Figure 1 shows the results of the systematic search strategy.

Table 1 summarizes the general characteristics of the studies. In total, 27 studies described employment in kidney failure patients during dialysis [17–43], 25 addressed employment after kidney transplantation [3, 4, 12, 13, 44–64], and 6 [14, 65–69] addressed both dialysis and kidney transplantation. In total, 33 studies regarding dialysis and 31 regarding kidney transplantation were included, with a total of 162,059 and 137,742 participants, respectively. The publication year of the included studies ranged from 1981 to 2020 (median: 2013). Most of the studies (81%) were cross-sectional in design, analysing data at a specific point in time. The cross-sectional studies [3, 12–14, 17–19, 21, 22, 24–39, 41–49, 51, 54, 55, 57, 59, 61–64, 66–69] were small to medium sized with a median of 139–233 participants for kidney transplant and dialysis patients, while the cohort studies [4, 20, 23, 40, 50, 52, 53, 56, 58, 60, 65] were mainly larger population studies (median of 2103 for dialysis patients and 1254 for kidney transplant patients). More than half of the studies were single-centre studies, and the studies were mainly from high-income countries. Study details are shown in Tables 1, 2 and 3.

**Table 1:** General characteristics of the included studies, by dialysis and kidney transplantion

Geography	Dialysis (*n* = 33)	Kidney transplantion (*n* = 31)
Europe	10	13
North America	11	14
Others (Asia, South America, New Zealand)	12	4
**Study design**		
Cross sectional	29	23
Cohort study	4	8
**Study sampling method**		
Single-centre	13	24
Multicentre	13	2
Registry	7	5
**Type of dialysis**^**a**^		
Haemodialysis	15	
Peritoneal dialysis	10	
Dialysis-modality unknown	17	
**Number of participants**		
*Cross sectional studies*		
Median	233	139
Range	43-105,636	34-1278
SD	22,449	255
*Cohort studies*		
Median	2103	1253
Range	359-4734	358-71,976
SD	1997	27,826

^a^Does not sum up to 33 because some studies included more than one type of dialysis

**Table 2:** Characteristics of the individual studies among kidney failure patients receiving dialysis

Reference	Country	Study design	Study population	Study period	Participation rate	Age years (mean)	Sex (Male) %	Results	Quality assessment
Albatineh 2019 [17]	Kuwait	Cross sectional	336 HD patients from six dialysis centres	n.a	n.a.	> 21	43.5	Employed 17.9%	4
Al -Jumaih 2011 [18]	Saudi Arabia	Cross sectional	100 HD patients selected randomly from 3 centres	n.a.	n.a.	(53.4)	68	Employed 28%	3
AlShahrani 2018 [19]	Saudi Arabia	Cross sectional	233 patients from all hemodialysis centres	2016-17	n.a.	> 20	78.5	Employed 26.6%	3
Curtin 1996 [39]	U.S.	Cross sectional	359 stratified from 31 centres	n.a.	n.a.	18-62 (43)	50	Employed: before dialysis 73%; during dialysis 24%	7
Ghani 2019 [20]	Sweden	Cohort	4734 patients; HD = 2667; PD = 2067	1995-2012	96%	HD/PD (48/47)	HD 65; PD 62	Employed: 1 yr before dialysis: total 65.3%; HD/PD 57%/76%; during dialysis: total 59.7%; HD/PD 51%/71%	6
Grubman-Nowak 2020 [69]	Poland	cross sectional	60 HD patients	2016-19	n.a.	(60)	60	Employed: 25%	3
Gutman 1981 [21]	U.S.	Cross sectional	2481 from 17 dialysis centres	1979	n.a.	21-59 (49)	55	Employed: 24%	8
Helanterá 2012 [14]	Finland	Cross sectional	819 from Finnish Kidney and Liver Association registry	2007	n.a.	15-64	62	Employed: total 23.9%; HD 19%; homeHD 44%; APD 39%; CAPD 16%	7
Holley 1994 [42]	U.S.	Cross sectional	77 patients; HD = 46; PD = 31	1993		21-54	47	Employed: 42.8%	5
Huang 2017 [22]	China	Cross sectional	166 patients in working age from 4 dialysis centres in Shanghai	2015	n.a.	(48.5)	64	Employed 15.7%	5
Imanishi 2017 [23]	Japan	Cohort	3151 dialysis patients in working age < 60	1999-2011	n.r.	18-59	n.a.	Employed 51%	5
Jarl 2018 [65]	Sweden	Cohort	1056 on dialysis from Swedish Kidney Registry	1995-2012	n.r.	20-60 (50.3)	63.5	Pre-dialysis:28%; during dialysis 18%	6
Julian Mauro 2013 [66]	Spain	Cross sectional	161 in dialysis (HD = 83; PD = 78) from 8 centres in Spain in working age	2007-9	n.a.	16-65 (41)	61.5	Employed: total 30.4%; HD 41%; PD 35.9%	3
Kasiske 1998 [24]	U.S.	Cross sectional	36,646 receiving dialysis placed on a waiting list for kidney transplant.	1994-96	n.r.	all ages	59	Employed pre-dialysis: Fulltime 53.4%; part-time 6.5%; during dialysis: Fulltime 34.5%; part-time 8.2%	5
Kutner 1991 [25]	U.S.	Cross sectional	283 dialysis patients, 15 patents from each of 81 treatment facilities	1987	99% of invited	18-59 (44.7)	n.a.	Employed 11%	4
Kutner 2008 [26]	U.S.	Cross sectional	105,636 dialysis patients from ESRD Facility Survey	2004	n.r.	18-54	n.a.	Employed 18.9%	6
Kutner 2010 [27]	U.S.	Cross sectional	1643 from US Renal Data System	2009	n.r.	> 18 (59.6)	55	Pre-dialysis 35.6%; During dialysis 11.6% (4 months after start)	5
Kwan 2013 [28]	Hong Kong	Cross sectional	All new consecutive automated PD-patients matched to CAPD-controls; 270; APD/CAPD 90/180	1995-2001	n.a.	APD/CAPD (50.5/57.8)	ADP 67; CAPD 54	Employed: Total 35.2%; APD/CAPD 71.2%/17%	5
Li 2018 [29]	Hong Kong	Cross sectional	101 (20 NHHD; 81 CAPD)	2009-14	87%	18-64 (47/52)	55	Employed: total 42.6%; NHHD: 80%; CAPD: 33.3%	4
Molsted 2004 [30]	Denmark	Cross sectional	112 from one university hospital; 59 in working age < 60 yr:	n.a.	75%	> 18 (57.8)	64	Employed (in working age): 22%	4
Nakayama 2015 [31]	Japan	Cross sectional	179 (102 PD; 77 HD) from 5 dialysis centres	2013	n.a.	(63)	68	Pre-dialysis: 63%; during dialysis 49.2%.	7
Neumann 2018 [32]	Germany	Cross sectional	353 (1 yr follow-up) stratified sample of 153 PD; 200 HD from 55 dialysis unit 6-24 months after initiation of dialysis	2014-2015	74%	> 18 (63.1)	68	Employed: total 17.1%; (PD 26.9%; HD 13.2%)	4
Panagopoulou 2009 [67]	Greece	Cross sectional	40 HD; 36 PD	n.a.	n.a.	HD/PD (57/59)	PD 58; HD 50	Employed before dialysis: HD: 78%; PD 43%; During dialysis: total 25%; HD: 20%; PD 31%	3
Parajuli 2016 [68]	U.S.	Cross sectional	200 from one kidney transplant center; dialysis > 1 yr before transplant	n.a.	48%	> 18 (57)	60	Employed before dialysis: HD 93.5% During dialysis HD 35%	4
Ravindran 2020 [43]	India	Cross sectional	503 HD patients from 11 centres	2015	95%	13-	74	Employed:11.1%	3
Takaki 2006 [33]	Japan	Cross sectional	317 HD patients from 4 dialysis centres	n.a.	n.a.	18-64 (54.2)	66	Employed: Total 42.3%; Male 54.1%; Female 19.4%	5
Tanaka 2020 [34]	Japan	Cross sectional	229; 36 PD + HD; 103 HD; 90 PD	2012-15	69.9%	PD + HD (57.4); HD (62.7); PD (65.5)	PD + HD 75; HD 80; PD 69	Employed: Total 52.8%; PD + HD 63.9%; HD 53.4%; PD 47.8%	4
Theorell 1991 [35]	Sweden	Cross sectional	470 patients in Sweden on dialysis	1988	65.5%	25-64	59.8	Employed: 20%	6
Walker 2016 [36]	New Zealand	Cross sectional	43; a part of a larger study	2014-15	n.a.	22-79	48	Employed: 27.9%	3
van Manen 2001 [40]	The Netherlands	Cohort	659 consecutive patients on dialysis; 359 completed follow-up	1997-99	54.5%	18-65 (48.7)	60	Employed: before dialysis 35%; 1 yr on dialysis: 29.8%	3
Wilk 2019 [37]	U.S.	Cross sectional	759 from one dialysis center	2010-18	65%	HD (59) INHD (50)	n.a.	Employed 10.5%	5
Wolcott 1988 [41]	U.S.	Cross sectional	33 PD; 33 HD matched by sex, age and diabetic status	n.a.	n.a.	20-65	70	Employed: 19.7% PD:30%; HD:9%	5
Zimmerman 2006 [38]	Canada	Cross sectional	81 patients randomly selected from a waiting list for donor transplant (1/3 not in dialysis)	n.a.	66%	(48.4)	56.2	Employed: 32.9%	4

*n.a* Not analyzed, *n.r.* Not relevant, *HD* Hemodialysis, *PD* Peritoneal dialysis, *yr* Year, *APD* Automate*d* Peritoneal Dialysis, *CAPD* Continuous Ambulatory Peritoneal Dialysis, *NHHD* Nocturnal home hemodialysis

**Table 3:** Characteristics of the individual studies among kidney failure patients receiving a kidney transplantion

Reference	Country	Study design	Study population	Study period	Participation rate (%)	Age years (mean)	Sex (Male) %	Results	Quality assessment
Bohlke 2008 [44]	Brazil	cross sectional	272 with kidney transplant-a systematic random sampling of 1512 kidney transplant patients from 11 centres stratified by transplantation centres	2003- 4	97%	> 18 (40.8)	n.a.	Pre-transplant employed: Full-time 11.8%; part-time 13.2%; Post-transplant employed: Full-time 23.2%; part-time 6.3%	9
Chen 2007 [45]	Taiwan	cross sectional	113 with kidney transplant	5 months (2003-4)	98%	> 18 (43.7)	54.9	Post-transplant employed: Full-time 50.4%; part-time 8%	3
Chisholm-Burnes 2012 [3]	U.S.	cross sectional	75 > 1 yr post-transplant	n.a.	90%	21-65 (47.6)	57.3	Post-transplant employed 39%	8
Danuser 2017 [4]	Switzerland	cohort	689 from the Swiss Transplant Cohort Study	2008-12	65%	18-65	65	Pre-transplant employed 58.9%; Post-transplant employed 56.2%	7
De Baere 2010 [46]	Belgium	cross sectional	79 with kidney transplant	n.a.	77.3%	18-65	62	Pre-transplant employed 63.1%; Post-transplant employed 58.6%	4
De Pasquale 2019 [47]	Italy	cross sectional	81 consecutive kidney transplant patients from one center	2016-17	72%	(46.3)	58	Pre-transplant employed 68%; Post-transplant employed 38%	5
Eng 2012 [12]	U.S.	cross sectional	204 with graft survival > 1 yr	2002-7	55%	18-65 (48.1)	57	Post-transplant employed 56%	7
Eppenberger 2015 [13]	Switzerland	cross sectional	354 with kidney transplant in one hospital; 282 in working age	2000-11	58%	42-61 (53.5)	71	Pre-transplant employed: Fulltime 33%; part-time 21%; 1 yr post-transplant: full-time 36%; part-time 20%	7
Grubman-Nowak 2020 [69]	Poland	cross sectional	101 patients with kidney transplant	2016-19	n.a.	(48)	60	Post-transplant employed 57%	3
Helanterá 2012 [14]	Finland	cohort	1818 with kidney transplant from Finnish Kidney and Liver Association registry	2007	n.r.	15-64 (49)	62	Post-transplant employed 40%	7
Jarl 2018 [65]	Sweden	cohort	3247 with kidney transplant from Swedish Kidney Registry	1995-2012	n.r.	20-60 (43.3)	64.5	Pre-transplant employed 62%; Post-transplant employed 61.1%	6
Jordakieva 2020 [48]	Austria	cross sectional	139 with kidney transplant in a multi-centre questionnaire study	2012	n.a.	18-55	58	Post-transplant employed: Full-time 36%; part-time 13.7%	5
Julian Mauro 2013 [66]	Spain	cross sectional	82 with kidney transplant from 8 centres in Spain in working age	2007-9	n.a.	16-65 (46)	58.5	Post-transplant employed: 39%	3
Markell 1997 [49]	U.S.	cross sectional	58 with kidney transplant patients from one outpatient clinic	1994	58%	20-67 (43)	50	Post-transplant employed: 43%	6
Matas 1996 [50]	U.S.	Cohort	636 with functioning kidney transplant	1985-1993	83%	> 18 (41)	62	Pre-transplant employed: Full-time 39%; part-time 5% Post-transplant employed: Full-time 32%; part-time 1%	5
Matas 2001 [551]	U.S.	Cross sectional	1278 with primary living donor kidney transplant	1990-98	n.a.	(32)	62	Post-transplant employed: Full-time 41%; part-time 4%	5
Messias 2014 [52]	Brazil	Cohort	358 with primary kidney transplants	2005-9	61.7	17-72 (37-49)	67	Post-transplant employed: 26%	6
Miyake 2019 [53]	Japan	Cohort	515 from one outpatient clinic being in paid employment at the time of transplant	2017-18	98%	20-64	68	Post-transplant employed: Full-time 76%; part-time 9%	5
Monroe 2005 [54]	U.S.	Cross sectional	78 with kidney transplant; in working age; a stratified sample from one center during a 10 yr period	n.a.	33%	23-62 (46.5)	52	Post-transplant employed 49%	4
Nour 2015 [55]	Canada	Cross sectional	60 with kidney transplant and functioning graft from one clinic	2003-8	41.7%	18-65 (52)	63.5	Pre-transplant employed 68.3%; Post-transplant employed 38.3%	6
Panagopoulou 2009 [67]	Greece	Cross sectional	124 patients with kidney failure and 48 with kidney transplant	n.a.	n.a.	(39)	67	Pre-transplant employed: 86%; Post-transplant employed 56%	3
Parajuli 2016 [68]	U.S.	Cross sectional	200 form one kidney transplant center; dialysis > 1 yr before transplant; investigated > 1 yr after transplant	n.a.	48%	28-82 (57)	60	Employed: Prior to dialysis 93.5%; during dialysis 35%; Post-transplant 35.5%	4
Petersen 2008 [56]	U.S.	Cohort	47,123 1 yr post kidney transplant from United States Renal Data System	1995-2002	n.r.	> 18 (45.9)	60	Employed: Pre-transplant: Fulltime 34.2%; part-time 6%; Post-transplant: Fulltime 38.1%; part-time 4.3%	7
Raiz 1997 [57]	U.S.	Cross sectional	180 with kidney transplant from one transplant center	n.a.	61.4%	> 19	53	Employed: Prior to kidney failure: 86%; Pre-transplant 53%; 1 yr post-transplant: 58%	8
Sangalli 2014 [58]	U.S	Cross sectional	227 with kidney transplant; in working age; 6 months follow-up from two outpatient clinics	2007-9	67%	18-65	59	Post-transplant employed: 56.5%	4
Slakey 2007 [59]	U.S	cross sectional	70 at least 48 months after kidney transplant; questionnaire study	1998-2000	47.9%	20-75 (47)	51	Post-transplant employed or in school 28.6%	4
Tzvetanov 2014 [60]	U.S.	Cohort	94,511 with kidney failure (baseline); *N* = 71,976 post-transplant from the United Network for Organ sharing database	2004-11	n.r.	18-64	n.a.	Employed pre-transplant: 33% 1 yr post-transplant 32.1%	6
van der Mei 2006 [61]	Netherlands	Cross sectional	239 with kidney transplant; 210 in working age	1996-2001	76.8%	19-71 (50.3)	n.a.	Employed:52.4%	5
van der Mei 2007 [62]	Netherlands	Cross sectional	61 (3-month post-transplant); 58 (1 yr post-transplant)	2002-3	79%	18-64 (44.2)	52.5	Employed: Pre-dialysis: 72%; 1 yr post-transplant: 52%;	5
van der Mei 2011 [63]	Netherlands	Cross sectional	34 (T3) from one outpatient clinic in paid employment at the time of transplant	2002-3	n.a.	18-64 (50.5)	55.9	Employed 6 yr post-transplant: 67%	5
Whitlock 2017 [64]	U.S.	Cross sectional	325 from one kidney transplant center	2011-15	n.a.	(52.3)	60.9	Post-transplant employed 14.2%	5

*n.a* Not analyzed, *n.r.* Not relevant, *yr* Year

### General description of study participants

The dialysis patients were on average 52.6 (16–79) years old, and the kidney transplant patients were 46.7 (18–78) years old. More than half of the dialysis and kidney transplant patients were males, 60.3 and 59.8%, respectively.

### Employment rate during dialysis and pre- and posttransplant

#### Before and during dialysis

The weighted mean for the employment rate during dialysis was 26.3% (range: 10.5–59.7), as shown in Tables 4 and 5. The employment rate was 21.6% in the 16 studies, which excluded patients more than 65 years of age [14, 20, 22, 23, 25, 26, 29, 30, 33, 35, 39–42, 65, 66, 69]. The U.S. generally appeared to have a lower employment rate among patients receiving dialysis treatment. Removing the studies conducted in the U.S. resulted in a weighted mean of 44.4% compared to 24.8% in the U.S. A total of 23 cross-sectional studies found an employment rate of 24.9%, compared to an employment rate of 51.7% in the 3 cohort studies.

**Table 4:** Employment rate in patients pre-dialysis and during dialysis, by continent (Weighted Mean, Standard deviation, SD, and Range)

Continent	Pre-dialysis			During Dialysis		
	Weighted mean (%)	SD	range	Weighted mean (%)	SD	range
Europe	57.1	16.7	28.0-65.3	45.8	12.3	17.1-59.7
North America	59.1	21.9	35.6-93.5	24.8	12.0	10.5-42.9
Other (Asia, South America, New Zealand)	63.0			41.4	14.3	11.1-52.8
**Total**	**59.0**	**22.0**	**28.0-93.5**	**26.3**	**13.5**	**10.5-59.7**

**Table 5:** Employment rate in patients pre- and post-kidney transplantation, by continent (Weighted Mean, SD, Range)

Continent	Pre-transplant			Post-transplant		
	Weighted mean (%)	SD	range	Weighted mean (%)	SD	range
Europe	61.3	11.1	54.0-86.0	53.7	8.9	38.0-67.0
North America	36.0	21.2	33.0-85.6	36.3	9.7	14.2-58.0
Other (Asia)	25.0			53.8	27.6	26.0-85.0
**Total**	**36.9**	**19.3**	**25.0-86.0**	**38.2**	**14.6**	**14.2-85.0**

In general, the employment rate decreased after the initiation of dialysis. In 9 studies, data before and after the initiation of dialysis were available [20, 24, 27, 31, 39, 40, 65, 67, 68]. In these studies, the employment rate decreased by 16.4% (weighted mean), ranging from a decrease of 5.2 to 58.5% within and between countries.

In a study from the U.S. of 1643 dialysis patients, 36% were employed before dialysis and 11.6% after the start of dialysis [27]. In a Japanese study, 63% were employed before dialysis and 49% after the start of dialysis; 50.7% of haemodialysis (HD) patients and 48% of peritoneal dialysis (PD) patients were employed [31].

Patients receiving PD had a higher employment rate, 58.8% [14, 20, 28, 29, 31, 32, 34, 39, 41, 42, 66, 67], than patients on HD, 39.5% [14, 17–20, 22, 23, 29–34, 37, 39, 41, 42, 66–69].

#### Pre- and posttransplant

The pretransplant employment rate was 36.9% (weighted mean), ranging from 25 to 86% between continents. The posttransplant employment rate was 38.2% (weighted mean, all studies), ranging between 14.2 and 85% within and between continents, as shown in Tables 4 and 5. The employment rate was 34.4% when including only the 18 studies of kidney transplant patients that excluded patients 65 years or more (i.e., those not of working age) [3, 4, 12–14, 46, 48, 49, 52–55, 58, 60, 61, 63, 65, 66]. In the 20 cross-sectional studies, the employment rate was 45% (weighted mean) compared to 37.1% (weighted mean) in 8 cohort studies (not significant).

In 14 studies, both pre- and posttransplant data were available [4, 13, 44, 46, 47, 50, 55, 57, 60, 62, 65, 67, 68]. In these studies, the change in the employment rate from pre- to posttransplant ranged from a decrease of 30% to an increase of 3.5%. The majority of the studies assessed the employment rate 1 year posttransplant. Only one study examined employment rates 1 and 5 years posttransplant, which were 38.1 and 35.6%, respectively (full-time work) [56].

A Swiss study including 354 patients identified 32.9% of patients working full-time 1 year before transplantation, 20.9% working part-time and 11.9% working part-time with partial disability pension; in total, 65.7% were employed. One year posttransplant, 36.2% worked full-time, 19.5% worked part-time, and 10.6% worked part-time with partial disability pension, for a total of 66.3% being employed [13]. Another Swiss study found approximately the same relatively high rate of employment pre- and posttransplant [4]. In a cohort study performed in the U.S. among 105,181 post-kidney transplant patients, 34.2% worked full-time, and 6% worked part-time pretransplant. One year posttransplant, 38.1% worked full-time, and 4.3% worked part-time [56]. In another U.S. study from 2014, among 27,981 kidney failure patients of working age (18–64 years), 33% worked pretransplant, and 32.1% worked 1 year posttransplant [60].

#### Dialysis versus posttransplant employment

The employment rate was 26.4% during dialysis (weighted mean) and 37.4% posttransplant (*p* < 0.0001). The difference remained significant when excluding data from U.S. but the employment rates were higher (44.4% vs. 53.6%). The posttransplant patients were on average slightly younger than the dialysis patients. The employment rate was 21.6% vs. 34.4% for dialysis and posttransplant patients, respectively, when we excluded patients 65 years or older (i.e., those not of working age). This supports a real difference between the groups.

### Predictors for employment during dialysis and posttransplant

#### During dialysis

Twelve studies had information on normative comparison data to use for meta-analysis of predictors for employment during dialysis, but for only a few of the predictors: dialysis modality (PD vs. HD), diabetes vs. nondiabetes, educational level (more than high school vs. high school or less), gender (male vs. female) and age [4, 20, 22, 23, 27, 33, 34, 39, 40, 42, 55, 58]. Predictors for employment during dialysis were not having diabetes, educational level greater than high school, peritoneal dialysis, and male gender. Heterogeneity was low among studies with nondiabetic patients, moderate among studies examining educational level and substantial/high among studies examining peritoneal dialysis and gender, as indicated by the I^2^ values (Table 6 and Figure 2a-e; Supplementary material). In three studies, age was available for analysis. Young age was also a predictor for employment, with a mean difference of − 2.68 (− 3.2–2.15) and I^2^ of 77%. Excluding low-quality studies from the meta-analysis did not significantly change the results but slightly increased the estimates.

**Table 6:** Predictors for employment during dialysis and post-transplant

	No of Studies	Participants	Heterogeneity			Meta-analysis	
			Chi^**2**^	***p***	I^**2**^ (%)	OR	(95% CI)
**Dialysis**							
Diabetes (non-diabetic/diabetic)	7	479	6.34	0.39	5%	1.68	(1.46, 1.93)
Education (>high school/<=high school)	6	1704	10.0	0.08	50%	2.57	(2.06, 3.21)
Dialysis modality (PD/HD)	6	6081	19.3	0.002	74%	2.24	(2.01, 2.51)
Gender (male/female)	6	215	128	< 0.001	96%	4.09	(3.59, 4.67)
**Post transplant**							
Gender (male/female)	12	253	13.1	0.29	16%	1.41	(1.19, 1.67)
Education (>high school/<=high school)	10	2139	11.9	0.22	24%	2.25	(1.85, 2.75)
Kidney donor (living donor /deceased donor)	10	2597	8.7	0.47	0%	2.74	(2.30, 3.27)
Pretransplant employed (employed/unemployed)	8	74,408	26.8	< 0.001	74%	13.63	(13.1, 14.2)
Diabetes (non-diabetic/diabetic)	8	3114	15.2	0.03	54%	1.62	(1.36, 1.92)
Ethnicity (white/other than white)	5	944	5.1	0.28	21%	1.95	(1.44, 2.64)
Age (< 50 yr/> = 50 yr)	5	1566	6.5	0.17	38%	2.29	(1.85, 2.84)
Dialysis modality (PD/HD)	4	749	2.7	0.45	0%	1.55	(1.02, 2.35)
Waiting time (< 2 yr/> = 2 yr)	4	1226	0.2	0.98	0%	1.82	(1.37, 2.42)
Depression (no depression/depression)	3	1084	2.2	0.33	9%	2.24	(1.53, 3.27)
Dialysis duration (< 2 yr/> = 2 yr)	2	477	3.2	0.08	68%	3.82	(2.51, 5.83)

#### Posttransplant

Fifteen of the studies reporting posttransplant employment rate also had information of normative comparison data to use for a meta-analysis of predictors for employment posttransplant [3, 4, 12, 13, 44, 48–52, 55, 58–60, 63, 69]. There were enough normative data for only some of the predictors: pretransplant employment, educational level, donor type, dialysis modality, diabetes, waiting time for transplant, time on dialysis, depression, gender, age, and ethnicity. The predictors for posttransplant employment with low heterogeneity were having a living donor, educational level more than high school, peritoneal dialysis, male gender, younger age, being white, waiting time for transplantation, and depression and with moderate heterogeneity were pretransplant employment, being without diabetes, and shorter time in dialysis (< 2 years) (Table 6 and Figure 3a-k; Supplementary). Excluding low-quality studies from the meta-analysis did not significantly change the results but slightly increased the estimates.

### Assessment of quality of included studies

The studies evaluating employment during dialysis were assessed as low quality (*n* = 8; 24.2%) [18, 19, 36, 40, 43, 66, 67, 69], medium quality (*n* = 20; 60.6%) [17, 20, 22–30, 32–35, 37, 41, 42, 65, 68], or high quality (*n* = 4; 12.1%) [14, 21, 31, 39].

Based on the Newcastle–Ottawa criteria of assessment, studies of posttransplant employment were assessed as low quality (score 1–3) (*n* = 4; 12.9%) [45, 66, 67, 69], medium quality (score 4–6) (*n* = 19; 61.3%) [46–55, 58–65, 68], or high quality (score 7–9) (*n* = 8; 25.8%) [3, 4, 12–14, 44, 56, 57].

Many studies were cross-sectional single-centre studies, with a relatively small number of participants and self-reported patient data. Only 3 studies were prospective cohort studies [4, 40, 50]. When including only the high-quality studies in the analyses, the employment for dialysis patients changed from 26.3% (weighted mean, all studies) to 25.2% (weighted mean, high-quality studies) (not significant). The posttransplant employment rate changed from 36.9% (weighted mean, all studies) to 42.5% (weighted mean, high-quality studies) (not significant). The quality assessment is shown in Supplementary Tables 7a–7d.

## Discussion

### Key findings

This is the first quantitative systematic review focusing on employment rates in kidney failure patients during chronic dialysis treatment and in patients receiving kidney transplantation. In the systematic review, we found that the employment rate considerably decreased during dialysis compared to predialysis, likely because the treatment constitutes a barrier to full- or part-time employment. However, the posttransplant employment rate decreased or increased only slightly compared to rates in the pretransplant and dialysis conditions. Our analyses support that it is very difficult to remain employed during dialysis and that employment depends on a combination of personal, clinical and work-related factors.

In the meta-analysis, the strongest predictor of posttransplant employment was shown to be pretransplant employment [4, 12, 13, 44, 49, 50, 52, 60], but there was high heterogeneity among studies. Danuser et al. found that 81% of patients who worked pretransplant were still employed posttransplant [4]. Sandhu et al. showed that among a U.S. population, employment gave privileged access to and shortened the waiting time for transplantation [70]. In the two prospective cohort studies [4, 50], employment status before transplant was also the most important predictor for employment 12 months after kidney transplant, which supports the results of this study and the result from Sandhu et al.

Educational level was also a predictor of posttransplant employment, as patients with a higher educational level were more likely to be employed posttransplant [3, 4, 12, 13, 44, 48, 55, 58, 59, 63]. Persons with a higher educational level may have more job opportunities and flexibility, lower physical workload, good insurance, and better health care, which may influence the possibilities for employment before kidney failure, during dialysis and posttransplant.

Being younger was also a predictor of posttransplant employment [4, 12, 13, 58, 59]. Danuser et al. found that younger patients were more likely to be employed before dialysis [4], which increased the chances of being in jobs during dialysis and posttransplant.

Having a living donor kidney may have also influenced employment status [3, 4, 12, 13, 44, 49, 50, 52, 55, 63]. However, the association of receiving a living donor kidney and posttransplant employment may not be causal but may depend to a greater extent on the resources of the recipient and their surroundings [71, 72]. Having diabetes and an ethnicity other than white were also associated with a lower rate of living donor kidney transplantation [4, 71, 73] and influenced employment levels [3, 4, 12, 44, 49–52, 58], supporting this assessment. A shorter waiting time for kidney transplantation increased the possibility of posttransplant employment [4, 12, 13, 55], which was shown especially for patients receiving a living donor kidney [4]. All these factors may therefore affect whether you receive a living donor and employment status. The differences in employment rates may also be explained by the fact that employment status determines the choice of dialysis modality and that employed patients with a higher level of education may have an increased interest and access to transplantation compared to unemployed patients [39, 40].

In general, employment constitutes a large and important part of our well-being and quality of life, and persons with high depression scores have lower well-being and quality of life and lower employment rates [4, 55, 58]. Studies have also shown that depression scores decreased in patients who were employed posttransplant [4, 44]. Therefore, less depression may be related to employment and not having a transplantation per se.

The employment rate for kidney failure patients differs between studies and countries, but in general, it is lower than the employment rate in the general population [8]. The variation between countries and continents may be related to differences in the mentioned predictors. Other factors may also have caused some of the differences, such as whether you have private or public health insurance. Kutner et al. in the U.S. showed that patients remaining employed after the initiation of dialysis were twice as likely to have employer-paid group insurance as those who did not remain employed [27]. Likewise, an Italian study by Sangalli et al. showed that employed individuals more often had private health insurance than unemployed individuals [58]. In contrast, a Chinese investigation found no effect on the employment level of having medical insurance [22]. Other studies have shown that the probability of returning to work is reduced if you already have a disability pension [49], but receiving a disability pension may also be explained by being more handicapped and potentially being unable to work. In countries without disability pensions, patients may either be forced to work, or they are dependent on support from their relatives.

This study has identified potential factors that may increase employment rates during dialysis and pre- and posttransplant, including maintenance of pretransplant employment. Educational support, support in maintaining a job during dialysis, and early return to work after transplantation seem important for posttransplant employment.

### Comparison with existing reviews

Only one earlier review investigated the employment rate posttransplant in all adult patients [7]. However, this review included only 9 studies and a population of only 23,059. They found an employment rate of 39.4% (weighted mean) posttransplant, while our review included 137,742 individuals with an employment rate of 38.2% (weighted mean, all studies) and 34.4% (weighted mean, only studies of patients below 65 years of age). The small differences in employment rates between the two reviews may be explained by the number of included studies and the large variation in employment rates between the individual studies.

A review of 16- to 30-year-old kidney failure patients showed that those on dialysis were more likely to be unemployed than patients having a kidney transplant, corresponding to the findings in our review [74]. Overall, the previous studies support the findings in the present study that dialysis and posttransplant patients have a lower employment rate than the general population.

### Strengths and limitations

The strengths of this review and meta-analysis are the wide search criteria ensuring inclusion of relevant studies and summarizing the knowledge of employment rate for kidney failure patients during dialysis and pre- and posttransplant. However, there are some limitations. First, nearly all studies had no control group and had no comparisons of employment rates with a background population. Second, most of the studies were cross-sectional in design, which limits the evidence of causality between employment and dialysis or kidney transplantation. Third, only a few studies had independent results of the employment rate, and many employment rates were self-reported, introducing a high risk of recall bias. Furthermore, 70% of the studies on dialysis and 45% of studies on kidney transplantation included subjects older than 65 years, which may have led to an underestimation of the real employment rate. However, excluding studies with patients > 65 years of age did not change the employment rate very much. Finally, many studies did not include all the relevant risk factors for unemployment. Moreover, each country has its own social laws and social and health insurance systems to support kidney failure patients staying at work or returning to work, which may have also influenced the employment rate, making it difficult to compare results across countries.

### Implications for future research and management of return to work

This review identified areas of concern among adults with kidney failure. However, caution is necessary regarding the limitations mentioned. As is the case for other diseases and health in general, kidney failure patients are also subject to social inequality regarding employment opportunities. There is a need for larger prospective cohort studies of kidney failure patients that ideally should include more detailed information about social and educational circumstances before and during replacement therapy and include comparisons of similar data with a relevant general background population from the same country.

Future studies should focus more on the predictors for staying employed to better understand the barriers and facilitation possibilities to support people with kidney failure to remain employed, including clarification of the importance of dialysis duration, time since diagnosis of severe chronic kidney disease, importance of family resources and specific social measures taken in each country. Future research should also focus on intervention through education, social support systems, and workplace and work task adaptation to find the best support systems to help kidney failure patients stay at work during dialysis and after transplantation. Additionally, studies should focus only on patients of working age with data on employment from independent sources such as tax or social benefits registries.

## Conclusion

Kidney failure patients have a low employment rate during dialysis and pre- and posttransplant. Predialysis employment, a higher education, not having diabetes or depression, being younger, male, or white, receiving a living donor kidney, and a short waiting time before transplantation were all predictors for posttransplant employment. It is important to support kidney failure patients through a combination of clinical and social measures to ensure that they remain in work.

## Supplementary Information

**Additional file 1: Table 7.a.** NEWCASTLE - OTTAWA QUALITY ASSESSMENT SCALE, NOS-score for Cross Sectional Studies. Dialysis^§^. **Table 7.b.** NEWCASTLE - OTTAWA QUALITY ASSESSMENT SCALE, NOS-score for Cohort Studies. Dialysis^§^. **Table 7.c.** NEWCASTLE - OTTAWA QUALITY ASSESSMENT SCALE, NOS-score for Cross Sectional studies. Pre- and Post-transplant^§^. **Table 7.d.** NEWCASTLE - OTTAWA QUALITY ASSESSMENT SCALE (NOS-score) for Cohort Studies. Pre- and Post-transplant^§^. **Figure 2.** a. Forest Plot of Comparison: Predictors for employment during dialysis. Outcome: Non-diabetic or Diabetic. b. Forest Plot of Comparison: Predictors for employment during dialysis. Outcome: Educational level more than high school or high school or less. c. Forest Plot of Comparison: Predictors for employment during dialysis. Outcome: Dialysis type: HD or PD. d. Forest Plot of Comparison: Predictors for employment during dialysis. Outcome: Gender: Male or Female. **Figure 3.** a. Forest Plot of Comparison: Predictors for post-transplant employment. Outcome: Gender: Male or Female. b. Forest Plot of Comparison: Predictors for post-transplant employment. Outcome: Educational Level; More Than High School or High School or Less. c. Forest Plot of Comparison: Predictors for post-transplant employment. Outcome: Living donor kidney or deceased donor. d. Forest Plot of Comparison: Predictors for post-transplant employment.

## Data Availability

The datasets used and/or analysed during the current study are available from the corresponding author upon reasonable request.

## Abbreviations

PD: Peritoneal dialysis
HD: Haemodialysis
APD: Automated peritoneal dialysis
CAPD: Continuous ambulatory peritoneal dialysis
NHHD: Nocturnal home
Yr: Year
NOS: Newcastle Ottawa Quality Assessment Scale
SD: Standard deviation
n.a.: Not analysed
n.r.: Not relevant
