# Supplementary material for: Employment of patients with kidney failure treated with dialysis or kidney transplantation—a systematic review and meta-analysis
Source: BMC Nephrol. 2021 Oct 22;22:348. doi: 10.1186/s12882-021-02552-2 (PMC8532382; doi:10.1186/s12882-021-02552-2)
Supplement: Supplementary file 1 — Additional file 1: Table 7.a. NEWCASTLE - OTTAWA QUALITY ASSESSMENT SCALE, NOS-score for Cross Sectional Studies. Dialysis§. Table 7.b. NEWCASTLE - OTTAWA QUALITY ASSESSMENT SCALE, NOS-score for Cohort Studies. Dialysis§. Table 7.c. NEWCASTLE - OTTAWA QUALITY ASSESSMENT SCALE, NOS-score for Cross Sectional studies. Pre- and Post-transplant§. Table 7.d. NEWCASTLE - OTTAWA QUALITY ASSESSMENT SCALE (NOS-score) for Cohort Studies. Pre- and Post-transplant§. Figure 2. a. Forest Plot of Comparison: Predictors for employment during dialysis. Outcome: Non-diabetic or Diabetic. b. Forest Plot of Comparison: Predictors for employment during dialysis. Outcome: Educational level more than high school or high school or less. c. Forest Plot of Comparison: Predictors for employment during dialysis. Outcome: Dialysis type: HD or PD. d. Forest Plot of Comparison: Predictors for employment during dialysis. Outcome: Gender: Male or Female. Figure 3. a. Forest Plot of Comparison: Predictors for post-transplant employment. Outcome: Gender: Male or Female. b. Forest Plot of Comparison: Predictors for post-transplant employment. Outcome: Educational Level; More Than High School or High School or Less. c. Forest Plot of Comparison: Predictors for post-transplant employment. Outcome: Living donor kidney or deceased donor. d. Forest Plot of Comparison: Predictors for post-transplant employment. [file 12882_2021_2552_MOESM1_ESM.zip › Figure 2a-e_predictors_dialysis 070921.pdf]

SUPPLEMENTARY MATERIAL

Figure 2.a. Forest plot of comparison: Predictors for employment during dialysis, outcome: Diabetes; non-diabetic or diabetic

	Employed		Unemployed			Odds Ratio	Odds Ratio
Study or Subgroup	Events	Total	Events	Total	Weight	M-H, Fixed, 95% CI	M-H, Fixed, 95% CI
Curtin 1996	67	85	202	274	6.5%	1.33 [0.74, 2.38]	
Holley 1994	28	33	31	44	1.3%	2.35 [0.74, 7.42]	
Huang 2017	4	26	19	140	1.6%	1.16 [0.36, 3.73]	
Imanishi 2017	1326	1617	1089	1534	64.9%	1.86 [1.57, 2.20]	
Kutner 2010	115	191	205	394	17.2%	1.40 [0.98, 1.98]	
Takaki 2006	151	184	107	133	7.2%	1.11 [0.63, 1.97]	
van Manen 2001	96	107	25	28	1.3%	1.05 [0.27, 4.04]	
**Total (95% CI)**		**2243**		**2547**	**100.0%**	**1.68 [1.46, 1.93]**	
Total events	1787		1678				

Heterogeneity: Chi² = 6.34, df = 6 (P = 0.39); I² = 5%

Test for overall effect: Z = 7.30 (P < 0.00001)

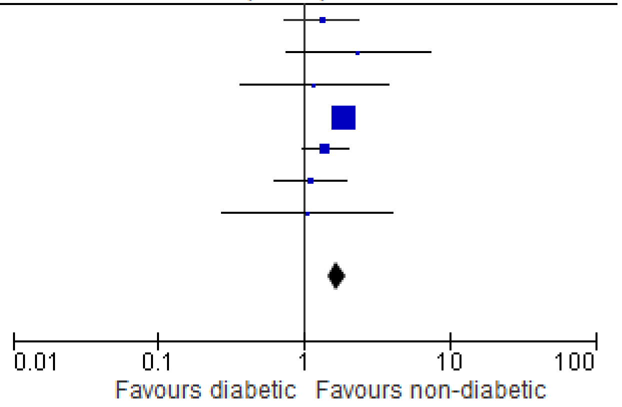

Figure 2.b. Forest plot of comparison: Predictors for employment during dialysis, outcome: Education; >high school or <high school

	Employed		Unemployed			Odds Ratio
**Study or Subgroup**	**Events**	**Total**	**Events**	**Total**	**Weight**	**M-H, Fixed, 95% CI**
Curtin 1996	49	85	79	274	16.1%	3.36 [2.03, 5.56]
Holley 1994	20	33	11	44	3.8%	4.62 [1.74, 12.25]
Huang 2017	31	117	6	114	4.5%	6.49 [2.59, 16.26]
Kutner 2010	124	191	175	394	40.7%	2.32 [1.62, 3.31]
Takaki 2006	79	184	39	133	26.2%	1.81 [1.13, 2.91]
van Manen 2001	66	107	14	28	8.6%	1.61 [0.70, 3.72]
**Total (95% CI)**		**717**		**987**	**100.0%**	**2.57 [2.06, 3.21]**
Total events	369		324			

Heterogeneity: $Chi^2 = 9.97$, $df = 5$ ($P = 0.08$); $I^2 = 50\%$

Test for overall effect: $Z = 8.32$ ($P < 0.00001$)

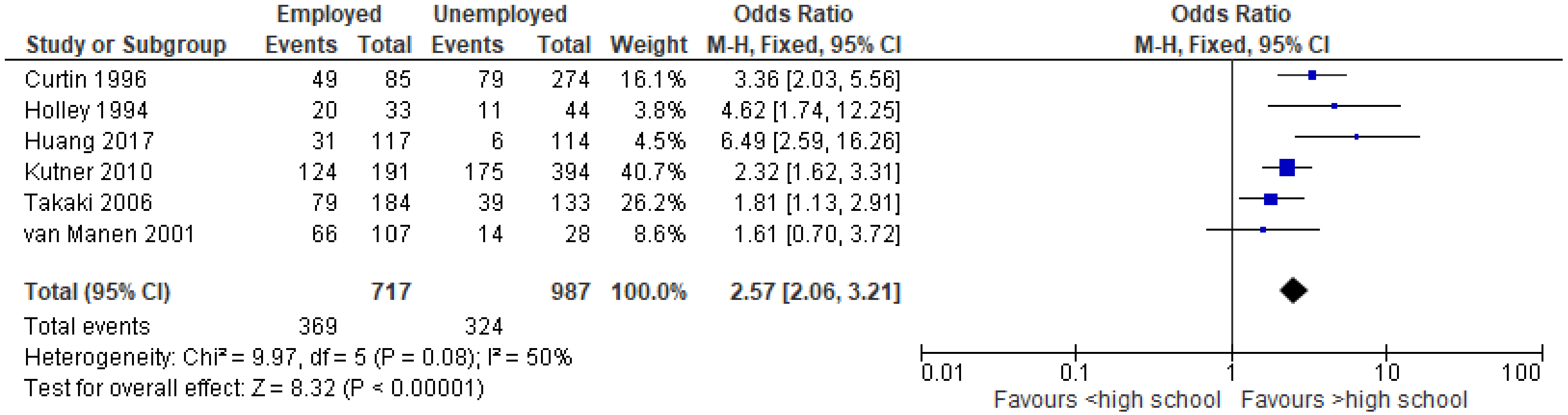

Figure 2.c. Forest plot of comparison: Predictors for employment during dialysis, outcome: Dialysis type; PD or HD

	Employed		Unemployed			Odds Ratio
Study or Subgroup	Events	Total	Events	Total	Weight	M-H, Fixed, 95% CI
Curtin 1996	12	85	30	271	3.0%	1.32 [0.64, 2.71]
Ghani 2019	1468	2828	599	1906	83.7%	2.36 [2.09, 2.66]
Holley 1994	16	33	15	44	1.6%	1.82 [0.72, 4.59]
Kutner 2010	36	191	23	394	3.0%	3.75 [2.15, 6.53]
Tanaka 2020	43	98	47	94	6.5%	0.78 [0.44, 1.38]
van Manen 2001	63	107	14	28	2.2%	1.43 [0.62, 3.30]
**Total (95% CI)**		**3342**		**2737**	**100.0%**	**2.23 [2.00, 2.50]**
Total events	1638		728			

Heterogeneity: $Chi^2 = 20.51$, $df = 5$ ($P = 0.001$); $I^2 = 76\%$
Test for overall effect: $Z = 14.02$ ($P < 0.00001$)

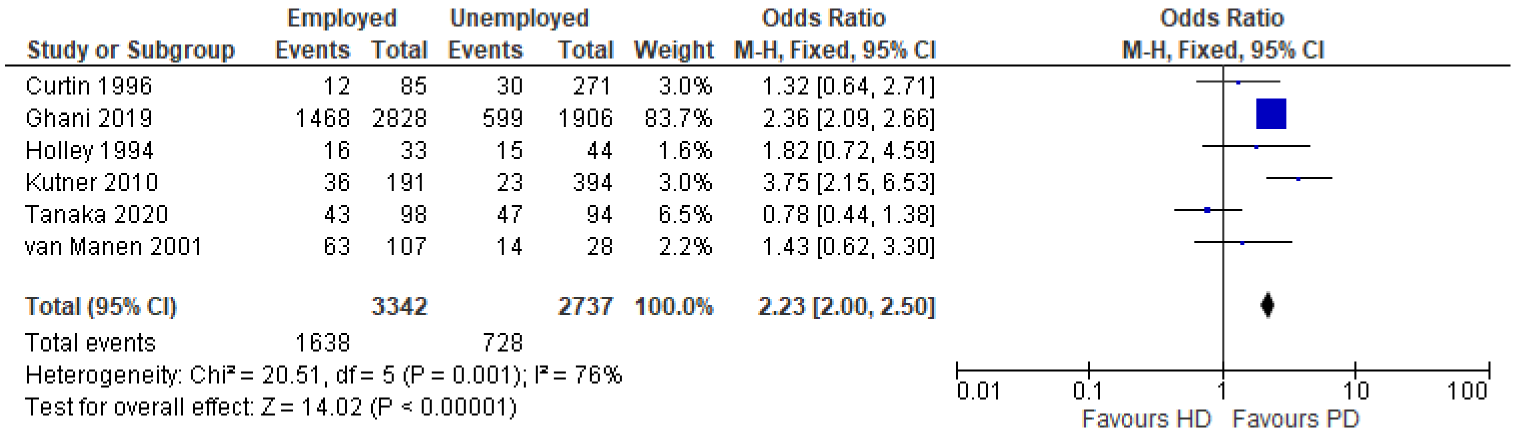

Figure 2.d. Forest plot of comparison: Predictors for employment during dialysis, outcome: Gender; Male or female

	Employed		Unemployed			Odds Ratio	Odds Ratio
**Study or Subgroup**	**Events**	**Total**	**Events**	**Total**	**Weight**	**M-H, Fixed, 95% CI**	**M-H, Fixed, 95% CI**
Curtin 1996	50	85	126	274	10.8%	1.68 [1.02, 2.75]	
Holley 1994	12	33	24	44	5.7%	0.48 [0.19, 1.20]	
Huang 2017	78	117	62	114	9.2%	1.68 [0.98, 2.86]	
Imanishi 2017	1342	1617	629	1534	48.1%	7.02 [5.95, 8.28]	
Kutner 2010	123	191	229	394	23.3%	1.30 [0.91, 1.86]	
van Manen 2001	84	107	19	28	2.8%	1.73 [0.69, 4.33]	
**Total (95% CI)**		**2150**		**2388**	**100.0%**	**4.09 [3.59, 4.67]**	
Total events	1689		1089				

Heterogeneity: Chi² = 128.04, df = 5 (P < 0.00001); I² = 96%

Test for overall effect: Z = 21.06 (P < 0.00001)

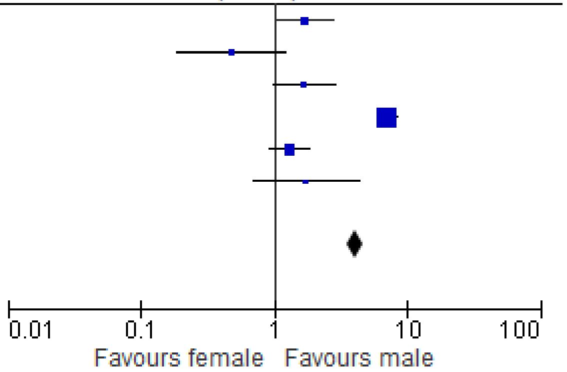

Figure 2.e. Forest plot of comparison: Predictors for employment during dialysis, outcome: Age; Young or older age

	Employed			Unemployed				Mean Difference	Mean Difference
Study or Subgroup	Mean	SD	Total	Mean	SD	Total	Weight	IV, Fixed, 95% CI	IV, Fixed, 95% CI
Huang 2017	44.55	8.89	26	49.41	7.4	140	2.1%	-4.86 [-8.49, -1.23]	
Imanishi 2017	48.4	8.3	1617	51.2	7.2	1534	92.9%	-2.80 [-3.34, -2.26]	
Kutner 2010	53.5	13.4	191	53	13.5	394	5.1%	0.50 [-1.82, 2.82]	
Takaki 2006	0	0	0	0	0	0		Not estimable	
**Total (95% CI)**			**1834**			**2068**	**100.0%**	**-2.68 [-3.20, -2.15]**	

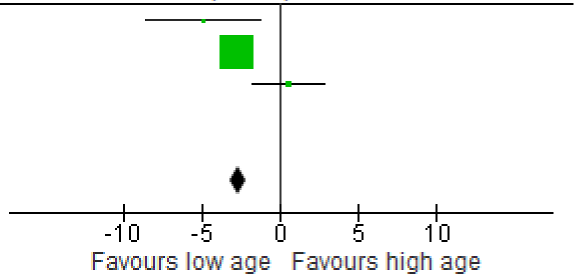

Heterogeneity: Chi² = 8.78, df = 2 (P = 0.01); I² = 77%
Test for overall effect: Z = 10.04 (P < 0.00001)
