# Supplementary material for: Employment of patients with kidney failure treated with dialysis or kidney transplantation—a systematic review and meta-analysis
Source: BMC Nephrol. 2021 Oct 22;22:348. doi: 10.1186/s12882-021-02552-2 (PMC8532382; doi:10.1186/s12882-021-02552-2)

SUPPLEMENTARY MATERIAL

Figure 3.a. Forest plot of comparison: Predictors for post-transplant employment, outcome: Gender; male or female

	Employed posttransplant		Unemployed posttransplant			Odds Ratio
**Study or Subgroup**	**Events**	**Total**	**Events**	**Total**	**Weight**	**M-H, Fixed, 95% CI**
Bohlke 2008	55	80	86	192	7.0%	2.71 [1.56, 4.71]
Chisholm-Burns 2011	17	25	26	46	2.6%	1.63 [0.59, 4.55]
Danuser 2017	259	387	188	302	30.7%	1.23 [0.90, 1.68]
Eng 2012	72	114	44	90	8.0%	1.79 [1.02, 3.14]
Eppenberger 2015	140	201	51	72	10.0%	0.95 [0.52, 1.71]
Grubman-Nowak 2020	39	58	22	43	3.6%	1.96 [0.87, 4.41]
Jordakleva 2020	41	72	39	67	7.7%	0.95 [0.48, 1.86]
Markell 1997	15	25	13	33	2.0%	2.31 [0.80, 6.67]
Messias 2014	53	78	146	220	10.8%	1.07 [0.62, 1.87]
Nour 2015	17	23	24	37	2.1%	1.53 [0.49, 4.85]
Sangalli 2014	121	190	77	145	14.0%	1.55 [1.00, 2.41]
van der Mei 2011	11	20	6	10	1.6%	0.81 [0.17, 3.81]
**Total (95% CI)**		**1273**		**1257**	**100.0%**	**1.41 [1.19, 1.67]**
Total events	840		722			

Heterogeneity: Chi² = 13.09, df = 11 (P = 0.29); I² = 16%

Test for overall effect: Z = 4.02 (P < 0.0001)

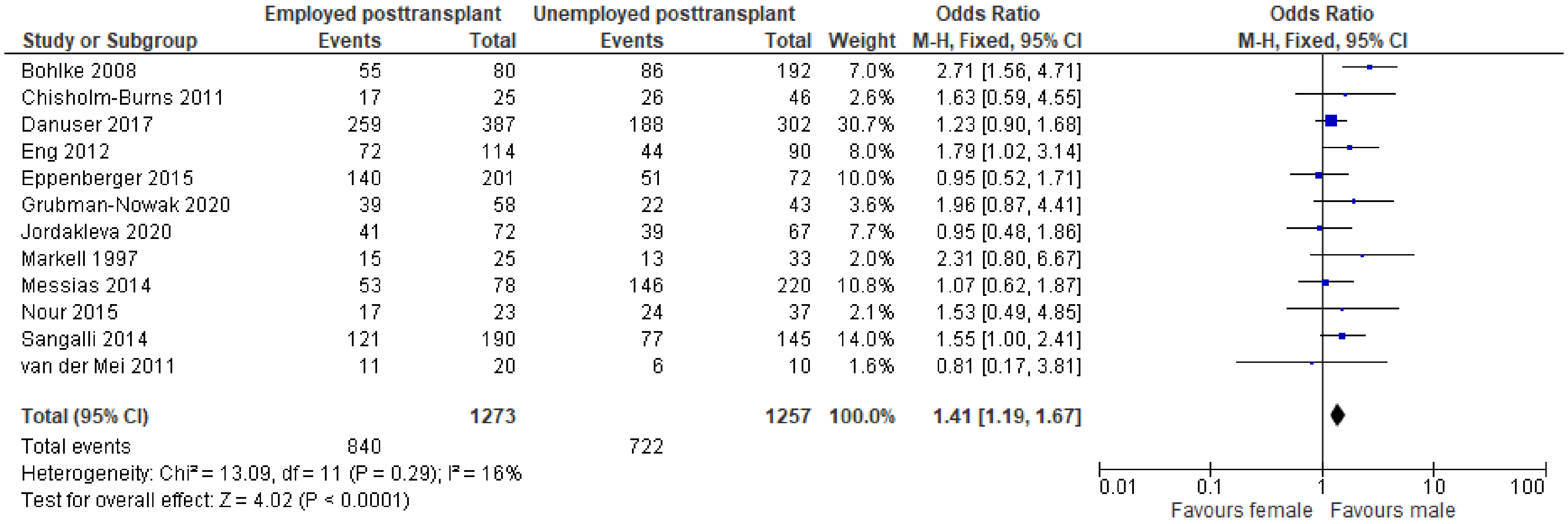

Figure 3.b. Forest plot of comparison: Predictors for post-transplant employment, outcome: Education; >high school or <high school

	Employed posttransplant		Unemployed posttransplant			Odds Ratio
**Study or Subgroup**	**Events**	**Total**	**Events**	**Total**	**Weight**	**M-H, Fixed, 95% CI**
Bohlke 2008	50	80	60	192	9.9%	3.67 [2.12, 6.33]
Chisholm-Burns 2011	25	29	28	46	2.2%	4.02 [1.20, 13.48]
Danuser 2017	131	387	62	302	34.4%	1.98 [1.40, 2.81]
Eng 2012	73	114	38	90	11.4%	2.44 [1.38, 4.29]
Eppenberger 2015	39	201	4	72	3.5%	4.09 [1.41, 11.90]
Jordakleva 2020	30	72	16	67	7.2%	2.28 [1.10, 4.73]
Nour 2015	18	23	17	37	2.1%	4.24 [1.30, 13.83]
Sangalli 2014	77	190	49	146	24.6%	1.35 [0.86, 2.11]
Slakey 2007	12	25	10	36	3.2%	2.40 [0.82, 7.01]
van der Mei 2011	14	20	5	10	1.5%	2.33 [0.49, 11.17]
**Total (95% CI)**		**1141**		**998**	**100.0%**	**2.25 [1.85, 2.75]**
Total events	469		289			

Heterogeneity: $Chi^2 = 11.85$, $df = 9$ ($P = 0.22$); $I^2 = 24\%$
Test for overall effect: $Z = 8.02$ ($P < 0.00001$)

Odds Ratio
M-H, Fixed, 95% CI

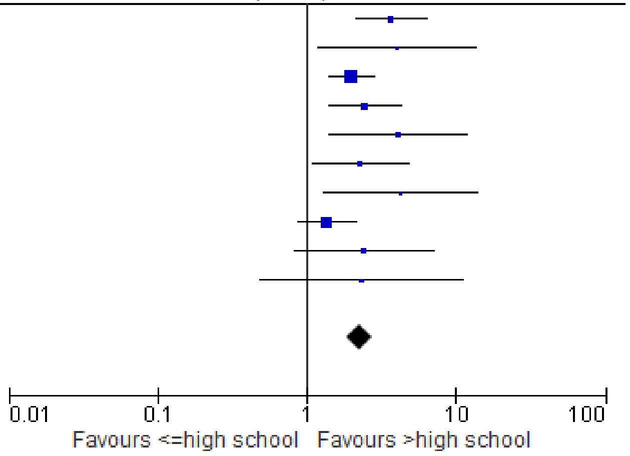

Figure 3.c. Forest plot of comparison: Predictors for post-transplant employment, outcome: Kidney donor; living or deceased donor

	Employed posttransplant		Unemployed posttransplant			Odds Ratio
Study or Subgroup	Events	Total	Events	Total	Weight	M-H, Fixed, 95% CI
Bohlke 2008	40	80	56	192	10.7%	2.43 [1.42, 4.16]
Chisholm-Burns 2011	14	29	12	46	3.1%	2.64 [0.99, 7.06]
Danuser 2017	206	387	84	302	28.6%	2.95 [2.14, 4.07]
Eng 2012	33	114	11	90	5.7%	2.93 [1.38, 6.19]
Eppenberger 2015	134	201	21	72	6.7%	4.86 [2.70, 8.73]
Markell 1997	5	25	4	33	1.8%	1.81 [0.43, 7.60]
Matas 1996	156	365	72	273	30.6%	2.08 [1.48, 2.93]
Messias 2014	46	78	62	220	8.6%	3.66 [2.14, 6.28]
Nour 2015	16	23	20	37	3.0%	1.94 [0.65, 5.83]
van der Mei 2011	6	20	2	10	1.2%	1.71 [0.28, 10.59]
**Total (95% CI)**		**1322**		**1275**	**100.0%**	**2.74 [2.30, 3.27]**
Total events	656		344			
Heterogeneity: Chi² = 8.66, df = 9 (P = 0.47); I² = 0%						
Test for overall effect: Z = 11.32 (P < 0.00001)						

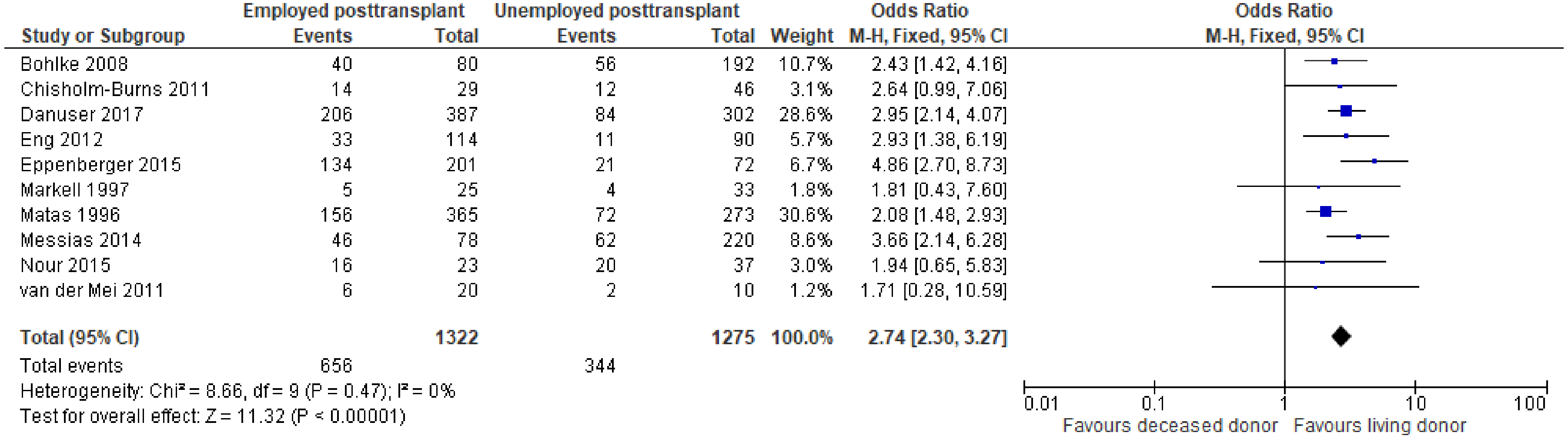

Figure 3.d. Forest plot of comparison: Predictors for post-transplant employment, outcome: Pretransplant employed; employed or not employed

Study or Subgroup	Employed posttransplant Events	Employed posttransplant Total	Unemployed posttransplant Events	Unemployed posttransplant Total	Weight	Odds Ratio M-H, Fixed, 95% CI
Bohlke 2008	36	80	32	192	0.7%	4.09 [2.29, 7.32]
Danuser 2017	332	387	74	302	0.8%	18.60 [12.62, 27.40]
Eng 2012	93	114	11	90	0.2%	31.81 [14.45, 69.99]
Eppenberger 2015	181	201	20	72	0.2%	23.53 [11.78, 47.02]
Markell 1997	24	25	23	33	0.1%	10.43 [1.24, 88.13]
Matas 1996	228	317	51	321	1.0%	13.56 [9.21, 19.97]
Messias 2014	50	78	19	220	0.2%	18.89 [9.77, 36.54]
Tzvetanov 2014	17961	27981	5123	43995	96.9%	13.60 [13.09, 14.13]
**Total (95% CI)**		**29183**		**45225**	**100.0%**	**13.63 [13.13, 14.15]**
Total events	18905		5353			

Heterogeneity: Chi² = 26.77, df = 7 (P = 0.0004); I² = 74%
Test for overall effect: Z = 136.89 (P < 0.00001)

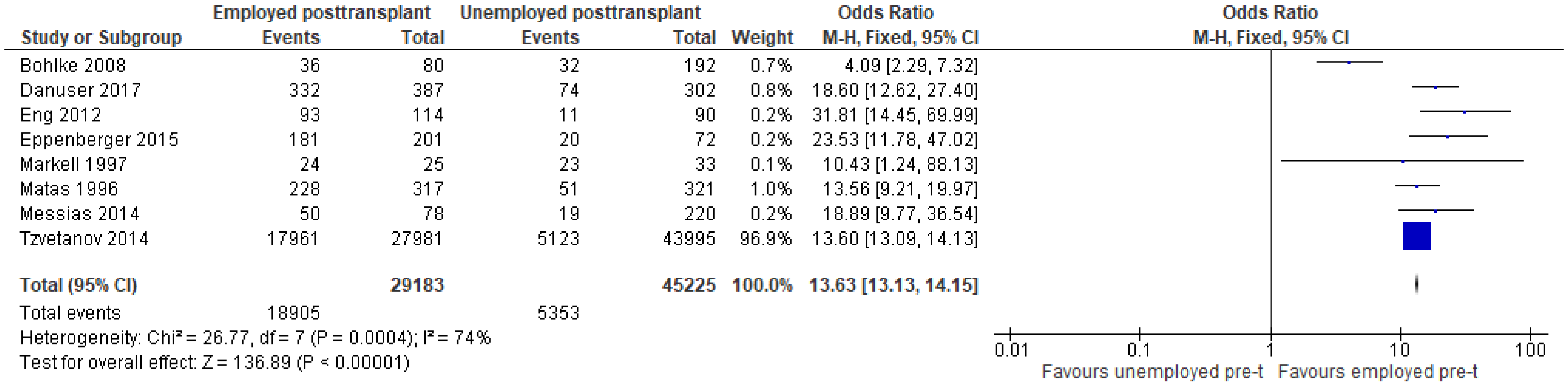

Figure 3.e. Forest plot of comparison: Predictors for post-transplant employment, outcome: Diabetes; non-diabetic or diabetic

	Employed posttransplant		Unemployed posttransplant			Odds Ratio
**Study or Subgroup**	**Events**	**Total**	**Events**	**Total**	**Weight**	**M-H, Fixed, 95% CI**
Bohlke 2008	73	80	149	192	3.7%	3.01 [1.29, 7.02]
Danuser 2017	350	387	250	302	13.1%	1.97 [1.25, 3.09]
Matas 1996	220	430	81	213	25.9%	1.71 [1.22, 2.39]
Matas 2001	220	500	120	286	41.8%	1.09 [0.81, 1.46]
Messias 2014	75	78	194	220	1.9%	3.35 [0.98, 11.40]
Nour 2015	19	23	31	37	2.0%	0.92 [0.23, 3.68]
Sangalli 2014	147	190	89	146	11.1%	2.19 [1.36, 3.52]
van der Mei 2011	18	20	6	10	0.4%	6.00 [0.87, 41.44]
**Total (95% CI)**		**1708**		**1406**	**100.0%**	**1.62 [1.36, 1.92]**
Total events	1122		920			

Heterogeneity: $Chi^2 = 15.24$, $df = 7$ ($P = 0.03$); $I^2 = 54\%$
Test for overall effect: $Z = 5.44$ ($P < 0.00001$)

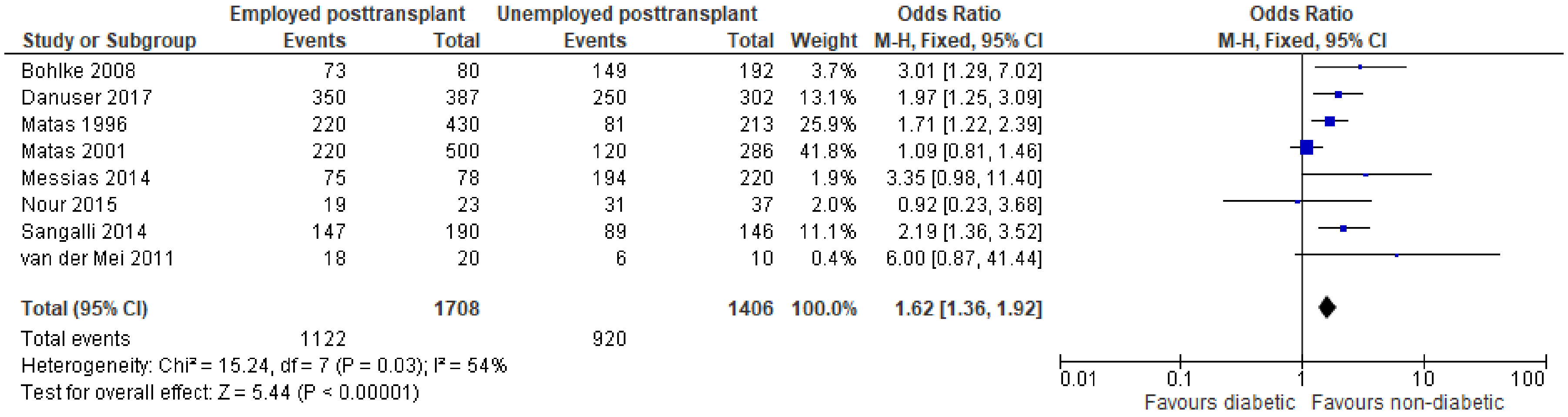

Figure 3.f. Forest plot of comparison: Predictors for post-transplant employment, outcome: Ethnicity; white or other than white

Study or Subgroup	Employed posttransplant Events	Employed posttransplant Total	Unemployed posttransplant Events	Unemployed posttransplant Total	Weight	Odds Ratio M-H, Fixed, 95% CI
Bohlke 2008	67	80	146	192	23.0%	1.62 [0.82, 3.21]
Chisholm-Burns 2011	19	29	15	46	6.6%	3.93 [1.47, 10.49]
Eng 2012	86	114	62	90	28.0%	1.39 [0.75, 2.57]
Markell 1997	11	25	5	33	4.0%	4.40 [1.28, 15.15]
Sangalli 2014	148	190	93	145	38.4%	1.97 [1.22, 3.19]
**Total (95% CI)**		**438**		**506**	**100.0%**	**1.95 [1.44, 2.64]**
Total events	331		321			

Heterogeneity: Chi² = 5.06, df = 4 (P = 0.28); I² = 21%
Test for overall effect: Z = 4.33 (P < 0.0001)

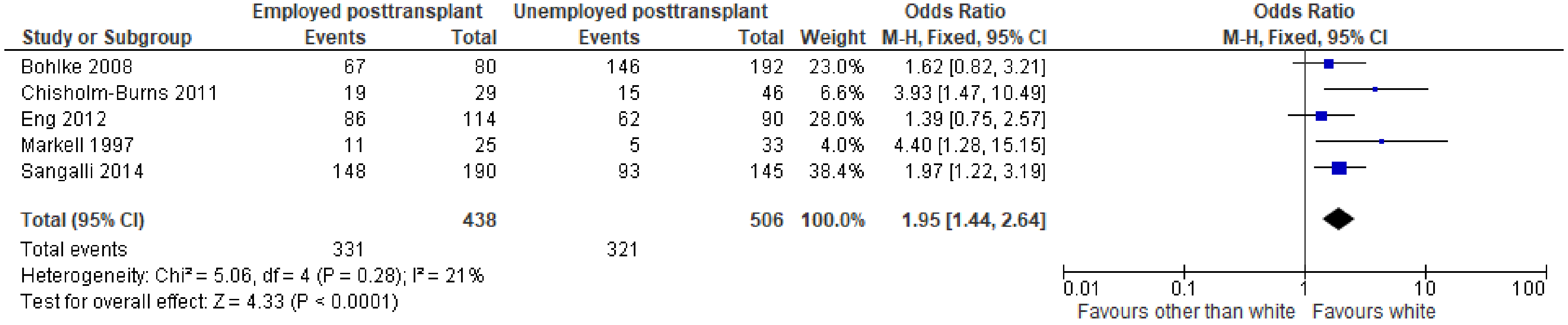

Figure 3.g. Forest plot of comparison: Predictors for post-transplant employment, outcome: Age; <=50 yr or >50 yr

	Employed posttransplant		Unemployed posttransplant			Odds Ratio
Study or Subgroup	Events	Total	Events	Total	Weight	M-H, Fixed, 95% CI
Danuser 2017	173	387	75	303	41.6%	2.46 [1.77, 3.41]
Eng 2012	79	114	37	90	11.4%	3.23 [1.81, 5.77]
Eppenberger 2015	119	201	29	72	15.6%	2.15 [1.24, 3.72]
Sangalli 2014	103	190	63	145	29.3%	1.54 [1.00, 2.38]
Slakey 2007	16	35	4	29	2.1%	5.26 [1.51, 18.32]
**Total (95% CI)**		**927**		**639**	**100.0%**	**2.29 [1.85, 2.84]**
Total events	490		208			

Heterogeneity: Chi^2 = 6.49, df = 4 (P = 0.17); I^2 = 38%

Test for overall effect: Z = 7.57 (P < 0.00001)

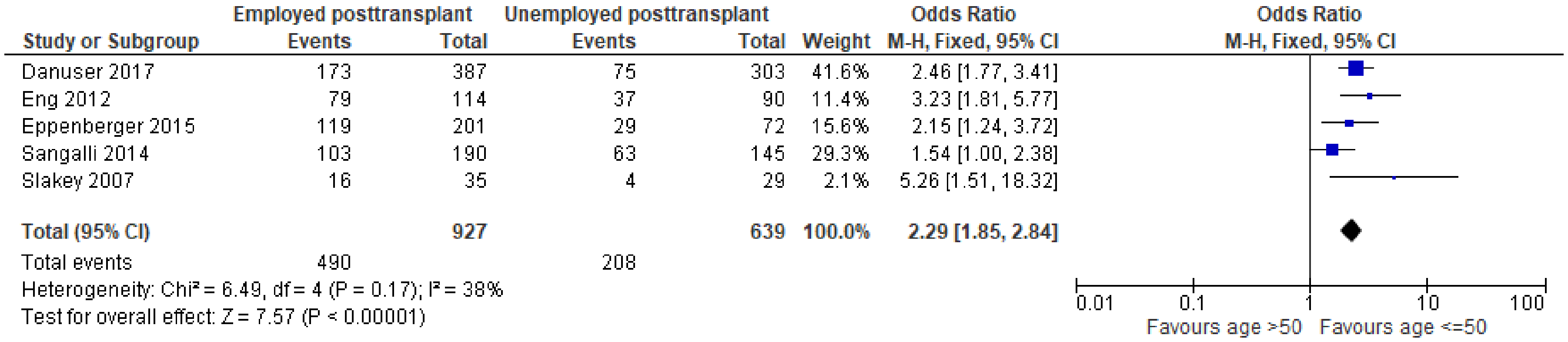

Figure 3.h. Forest plot of comparison: Predictors for post-transplant employment, outcome: Dialysis modality; peritoneal or haemodialysis

	Employed posttransplant		Unemployed posttransplant			Odds Ratio
Study or Subgroup	Events	Total	Events	Total	Weight	M-H, Fixed, 95% CI
Eng 2012	14	89	11	80	31.3%	1.17 [0.50, 2.75]
Markell 1997	2	23	5	32	12.3%	0.51 [0.09, 2.92]
Matas 1996	30	159	38	465	50.4%	2.61 [1.56, 4.38]
van der Mei 2011	16	20	7	10	6.0%	1.71 [0.30, 9.77]
**Total (95% CI)**		**291**		**587**	**100.0%**	**1.85 [1.22, 2.80]**
Total events	62		61			

Heterogeneity: Chi² = 4.91, df = 3 (P = 0.18); I² = 39%
Test for overall effect: Z = 2.90 (P = 0.004)

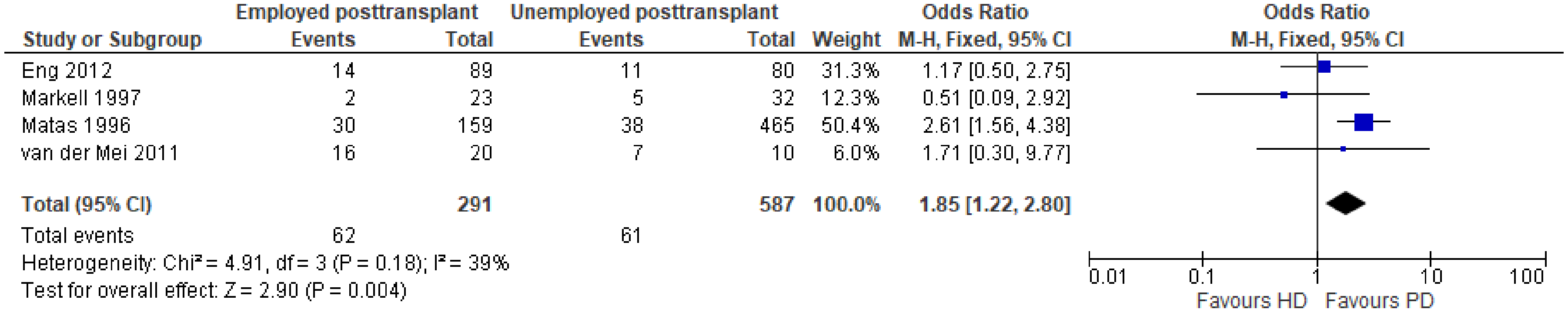

Figure 3.i. Forest plot of comparison: Predictors for post-transplant employment, outcome: Waiting time for transplant; <2 yr or >=2 yr

	Employed posttransplant		Unemployed posttransplant			Odds Ratio
**Study or Subgroup**	**Events**	**Total**	**Events**	**Total**	**Weight**	**M-H, Fixed, 95% CI**
Danuser 2017	330	387	232	302	53.1%	1.75 [1.19, 2.57]
Eng 2012	90	114	59	90	19.2%	1.97 [1.05, 3.68]
Eppenberger 2015	68	201	15	72	20.2%	1.94 [1.03, 3.68]
Nour 2015	14	23	18	37	7.5%	1.64 [0.57, 4.72]
**Total (95% CI)**		**725**		**501**	**100.0%**	**1.82 [1.37, 2.42]**
Total events	502		324			

Heterogeneity: $Chi^2 = 0.18$, df = 3 (P = 0.98); $I^2 = 0\%$

Test for overall effect: Z = 4.16 (P < 0.0001)

Odds Ratio
M-H, Fixed, 95% CI

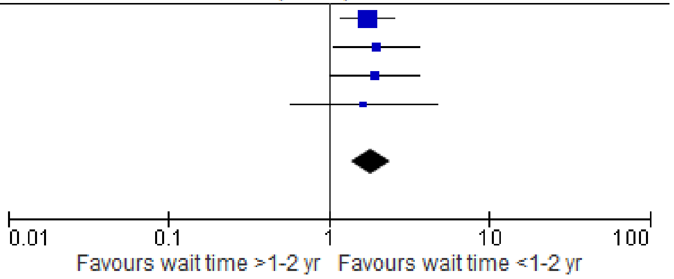

Figure 3.j. Forest plot of comparison: Predictors for post-transplant employment, outcome: Depression; no depression or depression

	Employed posttransplant		Unemployed posttransplant			Odds Ratio	Odds Ratio
**Study or Subgroup**	**Events**	**Total**	**Events**	**Total**	**Weight**	**M-H, Fixed, 95% CI**	**M-H, Fixed, 95% CI**
Danuser 2017	361	387	252	302	52.5%	2.75 [1.67, 4.54]	
Nour 2015	21	23	29	37	5.3%	2.90 [0.56, 15.05]	
Sangalli 2014	169	190	122	145	42.2%	1.52 [0.80, 2.87]	
**Total (95% CI)**		**600**		**484**	**100.0%**	**2.24 [1.53, 3.27]**	
Total events	551		403				
Heterogeneity: Chi² = 2.19, df = 2 (P = 0.33); I² = 9%							
Test for overall effect: Z = 4.17 (P < 0.0001)							

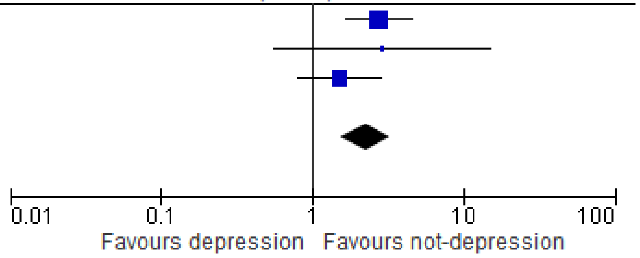

Figure 3.k. Forest plot of comparison: Predictors for post-transplant employment, outcome: Dialysis duration; <2yr or >=2 yr

Study or Subgroup	Employed posttransplant Events	Employed posttransplant Total	Unemployed posttransplant Events	Unemployed posttransplant Total	Weight	Odds Ratio M-H, Fixed, 95% CI
Eng 2012	62	114	28	90	61.9%	2.64 [1.48, 4.71]
Eppenberger 2015	121	201	15	72	38.1%	5.75 [3.05, 10.85]
**Total (95% CI)**		**315**		**162**	**100.0%**	**3.82 [2.51, 5.83]**
Total events	183		43			

Heterogeneity: $Chi^2 = 3.16$, $df = 1$ ($P = 0.08$); $I^2 = 68\%$
Test for overall effect: $Z = 6.22$ ($P < 0.00001$)

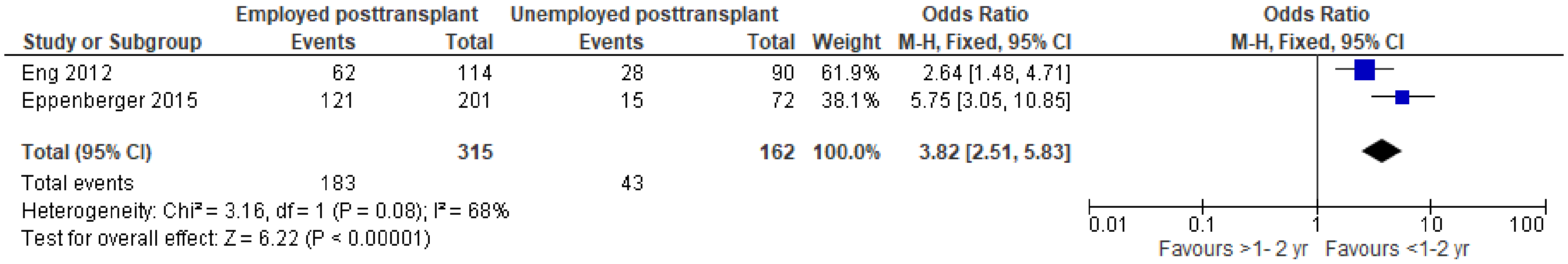

Supplement: Supplementary file 1 — Additional file 1: Table 7.a. NEWCASTLE - OTTAWA QUALITY ASSESSMENT SCALE, NOS-score for Cross Sectional Studies. Dialysis§. Table 7.b. NEWCASTLE - OTTAWA QUALITY ASSESSMENT SCALE, NOS-score for Cohort Studies. Dialysis§. Table 7.c. NEWCASTLE - OTTAWA QUALITY ASSESSMENT SCALE, NOS-score for Cross Sectional studies. Pre- and Post-transplant§. Table 7.d. NEWCASTLE - OTTAWA QUALITY ASSESSMENT SCALE (NOS-score) for Cohort Studies. Pre- and Post-transplant§. Figure 2. a. Forest Plot of Comparison: Predictors for employment during dialysis. Outcome: Non-diabetic or Diabetic. b. Forest Plot of Comparison: Predictors for employment during dialysis. Outcome: Educational level more than high school or high school or less. c. Forest Plot of Comparison: Predictors for employment during dialysis. Outcome: Dialysis type: HD or PD. d. Forest Plot of Comparison: Predictors for employment during dialysis. Outcome: Gender: Male or Female. Figure 3. a. Forest Plot of Comparison: Predictors for post-transplant employment. Outcome: Gender: Male or Female. b. Forest Plot of Comparison: Predictors for post-transplant employment. Outcome: Educational Level; More Than High School or High School or Less. c. Forest Plot of Comparison: Predictors for post-transplant employment. Outcome: Living donor kidney or deceased donor. d. Forest Plot of Comparison: Predictors for post-transplant employment. [file 12882_2021_2552_MOESM1_ESM.zip › Figure 3a-k_predictors transpl 070921.pdf]
