# Supplementary material for: Employment of patients with kidney failure treated with dialysis or kidney transplantation—a systematic review and meta-analysis
Source: BMC Nephrol. 2021 Oct 22;22:348. doi: 10.1186/s12882-021-02552-2 (PMC8532382; doi:10.1186/s12882-021-02552-2)
Supplement: Supplementary file 1 — Additional file 1: Table 7.a. NEWCASTLE - OTTAWA QUALITY ASSESSMENT SCALE, NOS-score for Cross Sectional Studies. Dialysis§. Table 7.b. NEWCASTLE - OTTAWA QUALITY ASSESSMENT SCALE, NOS-score for Cohort Studies. Dialysis§. Table 7.c. NEWCASTLE - OTTAWA QUALITY ASSESSMENT SCALE, NOS-score for Cross Sectional studies. Pre- and Post-transplant§. Table 7.d. NEWCASTLE - OTTAWA QUALITY ASSESSMENT SCALE (NOS-score) for Cohort Studies. Pre- and Post-transplant§. Figure 2. a. Forest Plot of Comparison: Predictors for employment during dialysis. Outcome: Non-diabetic or Diabetic. b. Forest Plot of Comparison: Predictors for employment during dialysis. Outcome: Educational level more than high school or high school or less. c. Forest Plot of Comparison: Predictors for employment during dialysis. Outcome: Dialysis type: HD or PD. d. Forest Plot of Comparison: Predictors for employment during dialysis. Outcome: Gender: Male or Female. Figure 3. a. Forest Plot of Comparison: Predictors for post-transplant employment. Outcome: Gender: Male or Female. b. Forest Plot of Comparison: Predictors for post-transplant employment. Outcome: Educational Level; More Than High School or High School or Less. c. Forest Plot of Comparison: Predictors for post-transplant employment. Outcome: Living donor kidney or deceased donor. d. Forest Plot of Comparison: Predictors for post-transplant employment. [file 12882_2021_2552_MOESM1_ESM.zip › Table 7a_d_suppl_mat.docx]

| **Table 7.a. NEWCASTLE - OTTAWA QUALITY ASSESSMENT SCALE, NOS-score for Cross Sectional Studies. Dialysis.^§^** | | | | | | | |  |  |
| --- | --- | --- | --- | --- | --- | --- | --- | --- | --- |
|  | **Selection:** | |  |  | **Comparability:** | **Outcome:** | | |  |
| References | 1) Representa­tiveness of the sample | 2) Sample size | 3) Non-respondents | 4) Ascertainment of the exposure (risk factor): | 1) The subjects in different outcome groups are comparable, based on the study design or analysis. Confounding factors are controlled. | 1) Assessment of the outcome: | 2) Statistical test: | | **Quality assessment NOS-score** |
| Albatineh et al, 2019 | * |  |  | ** |  | * |  | | 4 |
| AL-Jumaih et al, 2011 |  |  |  | ** |  | * |  | | 3 |
| Al-Shahrani et al, 2018 |  |  |  | ** |  | * |  | | 3 |
| Curtin et al, 1998 |  | * |  | ** | ** | * | * | | 7 |
| Grubman-Nowak 2020 |  |  |  | ** |  | * |  | | 3 |
| Gutman et al, 1981 | * | * |  | ** | ** | * | * | | 8 |
| Helanterá et al, 2012 | * | * | * | ** |  | * | * | | 7 |
| Holley et al, 1994 |  |  |  | ** | * | * | * | | 5 |
| Huang et al, 2017 |  |  |  | ** | * | * | * | | 5 |
| Julian-Mauro et al, 2013 |  |  |  | ** |  | * |  | | 3 |
| Kasiske 1998 | * | * |  | ** |  | * |  | | 5 |
| Kutner et al, 1991 | * |  |  | ** |  | * |  | | 4 |
| Kutner 2010 |  |  |  | ** | * | * | * | | 5 |
| Kutner 2008 | * | * | * | ** |  | * |  | | 6 |
| Kwan et al, 2013 | * |  | * | ** |  | * |  | | 5 |
| Li et al, 2018 | * |  |  | ** |  | * |  | | 4 |
| Molsted et al, 2004 | * |  |  | ** |  | * |  | | 4 |
| Nakayama et al, 2015 | * |  | * | ** | * | * | * | | 7 |
| Neumann et al, 2018 | * |  |  | ** |  | * |  | | 4 |
| Panagopoulou et al, 2009 |  |  |  | ** |  | * |  | | 3 |
| Parajuli et al, 2016 | * |  |  | ** |  | * |  | | 4 |
| Ravindran et al, 2020 |  |  |  | ** |  | * |  | |  |
| Takaki et al, 2006 |  |  |  | ** | * | * | * | | 5 |
| Tanaka et al, 2020 | * |  |  | ** |  | * |  | | 4 |
| Theorell et al, 1991 | * | * | * | ** |  | * |  | | 6 |
| Walker et al, 2016 |  |  |  | ** |  | * |  | | 3 |
| Wilk et al, 2019 | * | * |  | ** |  | * |  | | 5 |
| Wolcott et al, 1988 |  |  |  | ** | * | * | * | | 5 |
| Zimmerman et al, 2006 | * |  |  | ** |  | * |  | | 4 |

^§ A study can be given a maximum of one star (*) for each numbered item within the Selection categories (1-3) and Outcome categories. A maximum of two stars (**) can be given for Selection category 4 and for Comparability.^

| **Table 7.b. NEWCASTLE - OTTAWA QUALITY ASSESSMENT SCALE, NOS-score for Cohort Studies. Dialysis.** ^§^ | | | | | | | | |  | |
| --- | --- | --- | --- | --- | --- | --- | --- | --- | --- | --- |
|  | **Selection** |  |  |  | **Comparability** | **Outcome** |  |  | |  |
| References | 1) Representa-tiveness of the exposed cohort | 2) Selection of the non exposed cohort | 3) Ascertain-ment of exposure | 4) Demonstration that outcome of interest was not present at start of study | 1)  Comparability of cohorts on  the basis of the design or analysis | 1) Assessment of outcome | 2) Was follow-up long enough for outcomes to occur | 3) Adequacy of follow up of cohorts | | **Quality assessment NOS-score** |
| Ghani et al, 2018 | * |  | * | * |  | * | * | * | | 6 |
| Imanishi et al, 2017 | * |  | * | * |  |  | * | * | | 5 |
| Jarl et al, 2018 | * |  | * | * |  | * | * | * | | 6 |
| van Manen et al, 2001 | * |  | * |  |  |  | * |  | | 3 |

^§ A study can be given a maximum of one star for each numbered item within the Selection and Outcome categories. A maximum of two stars can be given for Comparability^

| **Table 7.c. NEWCASTLE - OTTAWA QUALITY ASSESSMENT SCALE, NOS-score for Cross Sectional studies. Pre- and Post-transplant.**^§^ | | | | | | | | |  |
| --- | --- | --- | --- | --- | --- | --- | --- | --- | --- |
|  | **Selection:** | | | | **Comparability:** | **Outcome:** | |  | |
| References | 1) Representativeness of the sample | 2)  Sample size | 3)  Non-respondents | 4) Ascertainment of the exposure (risk factor): | 1) The subjects in different outcome groups are comparable, based on the study design or analysis. Confounding factors are controlled. | 1) Assessment of the outcome: | 2) Statistical test: | **Quality assessment NOS-score** | |
| Bohlke 2008 | * | * | * | ** | ** | * | * | 9 | |
| Chen et al, 2007 |  |  |  | ** |  | * |  | 3 | |
| Chrisholm-Burns, 2011 | * |  | * | ** | ** | * | * | 8 | |
| De Baere, 2010 | * |  |  | ** |  | * |  | 4 | |
| De Pascale et al, 2019 |  |  |  | ** | * | * | * | 5 | |
| Eng et al, 2012 | * |  |  | ** | ** | * | * | 7 | |
| Eppenberger et al, 2015 | * |  |  | ** | ** | * | * | 7 | |
| Grubman-Nowak, 2020 |  |  |  | ** |  | * |  | 3 | |
| Helanterá et al, 2012 | * | * | * | ** |  | * | * | 7 | |
| Jordakieva et al, 2020 |  |  |  | ** | * | * | * | 5 | |
| Julian Mauro 2013 |  |  |  | ** |  | * |  | 3 | |
| Markell et al, 1997 |  |  |  | ** | ** | * | * | 6 | |
| Matas et al, 2001 | * | * |  | ** |  | * |  | 5 | |
| Monroe et al, 2005 | * |  |  | ** |  | * |  | 4 | |
| Nour et al, 2015 | * |  |  | ** | * | * | * | 6 | |
| Panagopoulou et al, 2009 |  | |  | ** |  | * |  | 3 | |
| Parajuli et al, 2016 | * |  |  | ** |  | * |  | 4 | |
| Raiz, 1997 | * | * |  | ** | ** | * | * | 8 | |
| Slakey et al, 2007 | * |  |  | ** |  | * |  | 4 | |
| van der Mei et al, 2011 | * |  | * | ** |  | * |  | 5 | |
| van der Mei et al, 2006 | * |  |  | ** | * | * |  | 5 | |
| van der Mei et al, 2007 | * |  | * | ** |  | * |  | 5 | |
| Whitlock et al, 2017 | * |  | * | ** |  | * |  | 5 | |

^§ A study can be given a maximum of one star (*) for each numbered item within the Selection categories (1-3)and Outcome categories. A maximum of two stars (**) can be given for Selection category 4 and for Comparability.^

| **Table 7.d. NEWCASTLE - OTTAWA QUALITY ASSESSMENT SCALE (NOS-score) for Cohort Studies. Pre- and Post-transplant.^§^** | | | | | | | | | | |  |
| --- | --- | --- | --- | --- | --- | --- | --- | --- | --- | --- | --- |
|  | **Selection** |  |  |  | **Comparability** | **Outcome** |  |  | |  | |
| References | 1) Representativeness of the exposed cohort | 2) Selection of the non exposed cohort | 3) Ascertainment of exposure | 4) Demonstration that outcome of interest was not present at start of study | 1)  Comparability of cohorts on the basis of the design or analysis | 1)  Assessment of outcome | 2)  Was follow-up long enough for outcomes to occur | 3)  Adequacy of follow up of cohorts | **Quality assessment NOS-score** | | |
| Danuser et al, 2017 | * |  | * | * | ** |  | * | * | 7 | | |
| Jarl et al, 2018 | * |  | * | * |  | * | * | * | 6 | | |
| Matas 1996 | * |  | * | * |  |  | * | * | 5 | | |
| Messias et al, 2014 | * |  | * | * |  | * | * | * | 6 | | |
| Miake et al, 2019 | * |  | * | * |  |  | * | * | 5 | | |
| Petersen et al, 2008 | * |  | * | * | ** |  | * | * | 7 | | |
| Sangalli et al, 2014 | * |  | * | * | * |  |  |  | 4 | | |
| Tzvetanov et al, 2014 | * |  | * | * |  | * | * | * | 6 | | |

^§^ A study can be given a maximum of one star for each numbered item within the Selection and Outcome categories. A maximum of two stars can be given for Comparability

***Supplementary:***

Figure 2.a. Forest Plot of Comparison: Predictors for employment during dialysis.

Outcome: Non-diabetic or Diabetic.

Figure 2.b. Forest Plot of Comparison: Predictors for employment during dialysis.

Outcome: Educational level more than high school or high school or less.

Figure 2.c. Forest Plot of Comparison: Predictors for employment during dialysis.

Outcome: Dialysis type: HD or PD.

Figure 2.d. Forest Plot of Comparison: Predictors for employment during dialysis.

Outcome: Gender: Male or Female.

Figure 3.a. Forest Plot of Comparison: Predictors for post-transplant employment.

Outcome: Gender: Male or Female.

Figure 3.b. Forest Plot of Comparison: Predictors for post-transplant employment.

Outcome: Educational Level; More Than High School or High School or Less.

Figure 3.c. Forest Plot of Comparison: Predictors for post-transplant employment.

Outcome: Living donor kidney or deceased donor.

Figure 3.d. Forest Plot of Comparison: Predictors for post-transplant employment.
